# PGC-1β and ERRα Promote Glutamine Metabolism and Colorectal Cancer Survival via Transcriptional Upregulation of PCK2

**DOI:** 10.3390/cancers14194879

**Published:** 2022-10-05

**Authors:** Danielle E. Frodyma, Thomas C. Troia, Chaitra Rao, Robert A. Svoboda, Jordan A. Berg, Dhananjay D. Shinde, Vinai C. Thomas, Robert E. Lewis, Kurt W. Fisher

**Affiliations:** 1 Eppley Institute, Fred & Pamela Buffett Cancer Center, University of Nebraska Medical Center, Omaha, NE 68198, USA; 2 Department of Pathology and Microbiology, University of Nebraska Medical Center, Omaha, NE 68198, USA; 3 Department of Biochemistry, University of Utah, Salt Lake City, UT 84112, USA

**Keywords:** PGC-1β, colorectal cancer, ERRα, PCK2, metabolism, K-Ras, precision medicine

## Abstract

**Simple Summary:**

Peroxisome Proliferator-Activated Receptor Gamma, Coactivator 1 Beta (PGC-1β) and Estrogen-Related Receptor Alpha (ERRα) are proteins that are over-expressed to support the survival of colorectal cancer (CRC) cells, but the details of how they promote the growth of CRC has not been defined. In this article, we determine that PGC-1β and ERRα work together to increase the transcription of mitochondrial Phosphoenolpyruvate Carboxykinase 2 (PCK2). We show that PCK2 is required by CRC cells to optimally use amino acid L-glutamine to generate energy through the TCA cycle to support tumor cell survival and this is one mechanism used by PGC-1β and ERRα to promote the growth of CRC.

**Abstract:**

Background: Previous studies have shown that Peroxisome Proliferator-Activated Receptor Gamma, Coactivator 1 Beta (PGC-1β) and Estrogen-Related Receptor Alpha (ERRα) are over-expressed in colorectal cancer and promote tumor survival. Methods: In this study, we use immunoprecipitation of epitope tagged endogenous PGC-1β and inducible PGC-1β mutants to show that amino acid motif LRELL on PGC-1β is responsible for the physical interaction with ERRα and promotes ERRα mRNA and protein expression. We use RNAsequencing to determine the genes regulated by both PGC-1β & ERRα and find that mitochondrial Phosphoenolpyruvate Carboxykinase 2 (PCK2) is the gene that decreased most significantly after depletion of both genes. Results: Depletion of PCK2 in colorectal cancer cells was sufficient to reduce anchorage-independent growth and inhibit glutamine utilization by the TCA cycle. Lastly, shRNA-mediated depletion of ERRα decreased anchorage-independent growth and glutamine metabolism, which could not be rescued by plasmid derived expression of PCK2. Discussion: These findings suggest that transcriptional control of PCK2 is one mechanism used by PGC-1β and ERRα to promote glutamine metabolism and colorectal cancer cell survival.

## 1. Introduction

PGC-1 family proteins (PGC-1α, PGC-1β, and PPRC1) are transcriptional co-activators that bind a diverse array of transcription factors to promote the transcription of genes that regulate metabolism [1]. The combinations of PGC-1 family members and transcription factors are highly context dependent. In the context of colon cancer, intestinal specific genetic deletion of Peroxisome Proliferator-Activated Receptor Gamma, Coactivator 1 Beta (PGC-1β) in mice does not harm normal colon epithelium, and makes the mice resistant to genetic and chemically induced carcinogenesis [2]. We have previously shown that PGC-1βand the transcription factor, Estrogen-Related Receptor Alpha (ERRα), are upregulated in colorectal cancer (CRC) in response to K-Ras mutations and that depletion of either protein decreases growth in vitro and in vivo [3,4]. However, the nature of the association between PGC-1β and ERRα and the genes they regulate in CRC has not been elucidated.

In this study, we first sought to confirm the interaction between PGC-1β and ERRα and subsequently identify the specific motif on PGC-1β required for the interaction with ERRα. Next, we identified the genes that are regulated by both PGC-1β and ERRα and found that mitochondrial Phosphoenolpyruvate Carboxykinase 2 (PCK2) was the gene most significantly decreased after depletion of either protein. Then, we explored the role of PCK2 in TCA cycle metabolism, glutamine utilization, and cell survival. Lastly, we determined if PCK2 can rescue the loss of ERRα on L-glutamine utilization and anchorage-independent growth.

## 2. Materials and Methods

### 2.1. Cell Culture

Colorectal cancer cell lines HCT116, T84, SW620, HT-29, SW480, and HCT15 were purchased from American Type Culture Collection (ATCC) and cultured in Dulbecco’s Modified Eagle’s Medium (DMEM) with 10% Fetal Bovine Serum (FBS), 2 mM L-glutamine and 1 mM sodium pyruvate at 37 °C with ambient oxygen (O2) and 5% CO2.

### 2.2. Lentiviral Transduction

For virus production, a 15-cm plate of HEK-293T cells at a confluence of 75% was transfected with 3 μg of pMD2.G, 6 μg of pPAX-2, and 12 μg of pLKO-shRNA-puro using 63 μL of polyethylenimine (PEI—1 μg/μL; Polysciences—24765) mixed in 750 μL of 10 mM HEPES pH 7.4 and 150 mM NaCl in water. The viral supernatant was cleared at 2000 RPM for five minutes before being filtered through a 0.45 μM membrane filter then centrifuged at 12,000 RPM for two hours in a Sorval Lynx6000 with a F14 rotor. The resulting pellet was resuspended in four mL of media and 8 μL of polybrene (8 μg/μL) was added. 1 mL of the virus-polybrene solution was mixed with 1 mL of media containing 500,000 colorectal cancer cells and plated in one well of a six well plate.

DNA sequences are listed in Supplementary Supplementary File S1.

### 2.3. Epitope-Tagging of Endogenous PGC-1β

The homology directed repair (HDR) template for epitope tagging of endogenous PGC-1β was prepared by Gibson assembly of 3 pieces: (1) A 5′ prime homology arm containing approximately 750 base pairs of the genomic DNA upstream of the PGC-1β stop codon, a tobacco etch virus (TEV) cleavage site, and a twin strep 2 epitope tag; (2) a central region containing a triple FLAG epitope and P2A-neomycin resistance cassette acquired via restriction endonuclease digestion of pFETCH_Donor (Addgene: 63934); and (3) a 3′ prime homology arm containing approximately 750 base pairs of the 3′ untranslated regions. Homology arms were ordered as gBlocks (Integrated DNA Technologies) and PCR-amplified. The three fragments were assembled with a Gibson Assembly Kit (New England Biolabs).

Then, HCT116 cells were transfected with the HDR template and pCAG-SpCas9-GFP-U6-gRNA (Addgene: 79144) expressing a gRNA targeted near the stop codon of PGC-1β. After two days, cells were moved to larger dishes, neomycin-selected, and clones were screened by PCR for correct genomic insertion.

DNA sequences are listed in Supplemental File S2.

### 2.4. Immunoprecipitation of PGC-1β

Sample Preparation: Twenty 15-cm cell culture dishes of HCT116 or T84 cells expressing epitope tagged PGC-1β with 75% confluence were washed with PBS and each dish was lysed in 300 μL of RIPA lysis buffer (1% Triton X-100) with protease and phosphatase inhibitors (Halt Cocktail). Cells were sonicated and cleared in a centrifuge at 4 °C for 20 min at 13,000 RPM in Thermo-Scientific Sorvall Lynx 6000 Centrifuge with F14 fixed angle rotor. Protein concentration was determined by BCA and samples were normalized to the same volume and concentration with additional RIPA buffer.

FLAG Immunoprecipitation: 200 μL of 50:50 magnetic FLAG bead slurry (Millipore-Simga, Burlington, MA, USA; M8823) were washed twice with Tris Buffer Saline (TBS) and added to each sample and rotated overnight at 4 °C. The beads were collected on a magnet and washed four times in 1 mL of TBS and eluted for three hours in 100 μL of 3X-FLAG peptide (100 ng/µL) in water.

Strep2 Immunoprecipitation: 100 μL of a slurry of MagStrep “type3” XT beads (IBA biosciences, 2-4090-010) were washed in TBS and added to each sample and rotated overnight at 4°. The beads were collected on a magnet and washed four times in 1 mL of Tris Buffer Saline (TBS) and eluted for three hours in 100 μL of Buffer BXT (0.1 M Tris-Cl, 150 mM NaCl, 1 mM EDTA, 50 mM biotin, pH 8).

### 2.5. Inducible PGC-1β Vector and Cell Line Generation

A full length human PGC-1β cDNA (Kind gift of Donald McDonnell, Duke) was PCR-amplified, digested, and ligated into AAVS1_Puro_Tet3G_3xFLAG_Twin_Strep (Addgene: 92099), and verified with bidirectional Sanger sequencing. Site directed mutagenesis was performed with the QuikChange Lightning Site-Directed Mutagenesis Kit (Agilent; 210513). Plasmids were integrated via dual transfection with pCAG-SpCas9-GFP-U6-gRNA (Addgene: 79144) expressing a gRNA targeting the AAVS1 T2 site.

DNA sequences for mutagenesis reactions are listed in Supplemental File S3.

### 2.6. siRNA Transfections

The siRNA oligos (Dharmacon, Lafayette, CO, USA) targeting PGC-1β, ERRα, PCK2 or non-targeting controls were used for targeted depletion of the colorectal cancer cells. For pooled transfections, two validated, individual ON-TARGET PLUS siRNAs were used at a final RNAi concentration of 40 nM and were added to 5 µL of RNAiMAX (ThermoFisher, Waltham, MA, USA, 13778150) and 500 µL Hank Buffer Salt Solution without sodium bicarb. The mixture was added to 300,000 cells in 1.5–2 mL of media without antibiotics in a 6-well plate. All transfections were conducted for 72-h before analysis.

RNA sequences for transfections are listed in Supplemental File S4.

### 2.7. RNA Sequencing and Analysis

RNA sequencing (RNA-seq) analysis was conducted by the UNMC Genomics Core. Cells were harvested using 0.5 mL TRIzol (ThermoFisher Scientific) and stored at −80 °C until RNA extraction was performed. RNA was extracted using RNeasy spin columns (Qiagen, Hilden, Germany) per manufacturer’s protocol. Final RNA was eluted with nuclease-free water and quantified using the NanoDrop 2000 (ThermoFisher Scientific). Three biological replicates of non-targeting control, PGC-1𝛽, or ERR𝛼 knockdown were completed using two separate siRNA oligos for each condition. Unstranded (poly A only) RNA sequencing libraries and 500 ng of total RNA for each of the samples were prepared per manufacturer’s suggested protocol using the TrueSeq mRNA Protocol Kit (Illumina, San Diego, CA, USA). Purified libraries were pooled at a 0.9 pM concentration and sequenced on an Illumina NextSeq550 instrument and 75 bp paired end sequencing was performed. Libraries were normalized and equal volumes were pooled in preparation for sequence analysis. Raw sequence data has been deposited as GSE147905 in the National Center for Biotechnology Information Gene Expression Omnibus. Sequence reads were preprocessed using XPRESSpipe (v0.4.1) [5], with adapter sequences AGATCGGAAGAGCACACGTCTGAACTCCAGTCA and AGATCGGAAGAGCGTCGTGTAGGGAAAGAGTGT. Reads were processed using H. sapiens GRCh38.13 Ensembl release 99. Differential expression analysis was performed using XPRESSpipe wrapper for DESeq2 (v1.22.1) [6]. Differentially expressed genes were further visualized using XPRESSplot. Isoform abundance analysis was performed using XPRESSpipe wrapper for Cufflinks (v2.1.1) [7] and IGV (v2.4.19) [8]. Scripts used to perform these analyses can be found at https://github.com/j-berg/frodyma_2020 (accessed on 31 March 2020).

### 2.8. Western Blot Analyses

Whole cell lysate extracts were prepared in radioimmunoprecipitation assay (RIPA) buffer that was comprised of 50 mM Tris-HCl, 1% NP-40, 0.5% sodium deoxycholate, 0.1% sodium dodecyl sulfate, 150 mM NaCl, 2 mM EDTA, 2 mM EGTA, and addition of a protease and phosphatase inhibitor cocktail (Halt, ThermoFisher Scientific). A BCA protein assay (Promega) was used to determine protein concentration. An 8% Acrylamide SDS-PAGE was used to separate out the protein and nitrocellulose membranes were blocked in Odyssey TBS blocking buffer (LI-COR Biosciences) for at least 30 min at room temperature. The primary antibody was allowed to hybridize at least overnight at 4 °C. The PCK2 (8565) antibody was obtained from Cell Signaling Technologies and used at a concentration of 1:2000. The PGC-1β (NBP1-28722) antibody was purchased from NovusBio and used at a concentration of 1:1000. The ERRα (ab76228) antibody was purchased from Abcam and used at a concentration of 1:1000. The FLAG epitope (F1804) antibody was purchased from Millipore-Sigma and used at a concentration of 1:5000. The Strep2 epitope (Ab02208-1.1) antibody was purchased from Absolute Antibody and used at a concentration of 1:2000. The β-actin (sc-47778) and α-tubulin (sc-5286) antibodies were purchased from Santa Cruz Biotechnology and used at a concentration of 1:2000. The anti-ALFA recombinant nanobody-rabbit Fc fusion (N1583) was obtained for NanoTag Biotechnologies and used at a concentration of 1:2000. IRDye 800CW and 680RD secondary antibodies (LI-COR Biosciences, Lincoln, NE, USA) were diluted 1:10,000 in 0.1% TBS-Tween and imaged on an Odyssey Scanner (LI-COR Biosciences).

Original images for Western Blot figures are provided in Supplemental File S5.

### 2.9. L-Glutamine Utilization Assays

The Seahorse XFe96 Metabolic Flux Analyzer (Agilent) was used to measure Oxygen Consumption Rate (OCR) in the presence of only 2 mM L-glutamine as a substrate. The day before the experiment, the FluxPak plates were hydrated in water and incubated at 37 °C with ambient CO2. The afternoon before the assay, 40,000 cells were plated in each well of a 96 well assay plate in 12 replicates in regular media. On the day of the experiment, the media was removed and the cells were washed twice with 1 mL of PBS and then covered in 180 µL of XF DMEM medium pH 7.4, (Agilent 103575-100) with 2 mM glutamine (Agilent 103579-100). The cells were incubated in this media at 37 °C with ambient CO2 for 1 h prior to beginning the experiment. The water was removed from the FluxPak and calibration media was added and incubated at 37 °C with the ambient atmosphere for an hour prior to the experiment. Basal OCR was measured four times for three minutes with mixing between measurements to ensure stability and the last measurement was used for statistical evaluation.

### 2.10. Intracellular Metabolite Analysis

HCT116 cells were transfected in five biological replicates, as previously described. 72-h after transfection, the cells were harvested and counted. After washing in saline solution, the cell pellet was resuspended into 1 mL of ice-cold 2:2:1 MeOH: ACN: H2O (*v/v/v*) containing 10 μM stable isotope-labeled canonical amino acid mix (Cambridge Isotope Laboratories, Inc., Tewksbury, MA, USA) as internal standards. The cells were subsequently lysed in a reciprocal shaker with 0.1 mm glass beads and the samples were centrifuged for 15 min at 13,000 rpm at 4 °C. The supernatant was removed and evaporated to dryness in the SpeedVac. The samples were reconstituted in 100 µL of resuspension buffer containing 20% ACN and 10 mM ammonium acetate, before LC-MS/MS analysis.

Chromatographic separation and mass Spectrometry detection were performed using a Shimadzu Nexera ultra-high-performance liquid chromatography (UHPLC) and triple-quadrupole-ion trap hybrid Mass spectrometer (QTRAP 6500 from Sciex, Framingham, MA, USA), equipped with an ESI source. The chromatographic separation of metabolites was achieved on a XSelect (150 × 2.0 mm id; particle size 1.7 µm) analytical column maintained at 40 °C. The optimum mobile phase consisted of 10 mM tributylamine with 5 mM acetic acid in LC-MS grade water containing 2% isopropanol as buffer A and isopropanol as solvent B. The gradient elution is performed as: time zero to five min, 0% solvent B; next 4 min, 2% solvent B; 0.5 min, 6% solvent B; 2 min, 6% solvent B; for next 0.5 min, solvent B was increased to 11% and maintained for 1.5 min; at 35 min, solvent B was increased to 28% for next 2 min and then to 53% in 1 min and maintained for next 6.5 min. Solvent B was reduced to 0% and maintained to equilibrate column till the next injection. The flow rate was 0.4 mL/min, and the total run time was 33 min, and the autosampler temperature was 10 °C. The data acquisition was under the control of MultiQuant software (Sciex, USA). The mass spectrometer was operated in positive as well as negative ion mode using polarity switching. Ions were acquired in multiple reaction monitoring (MRM) mode. MRM details for the selected metabolites were as follows: Oxaloacetate, 131.0/87.0; Phosphoenolpyruvate, 167.0/79.0; Citrate/Isocitrate pool, 191.0/111.0; Fumarate, 115.0/71.0; Succinate, 117.0/99.0. The retention time of each metabolite was confirmed by the 13C-labelled yeast metabolite extract, which was used as the qualitative standard (Cambridge Isotope Laboratories, Inc.). Optimized spray voltage was at 5.5 kV for positive and 4.2 kV for negative mode, ESI source temperature was at 400 °C, nitrogen was used as curtain gas, gas 1 and gas 2 at pressure 30, 40 and 40 arbitrary units, respectively. Declustering potential in positive and negative modes was optimized at 65 and −65 volts.

### 2.11. PCK2-ALFA Plasmid for Stable Expression

A full length PCK2 cDNA was obtained through Addgene (plasmid: 23715) and was PCR amplified with a 3′ primer containing a single ALFA tag. The resulting PCR product was digested and ligated into pcDNA-hEF-1α-neomycin resistance. The final product was verified with Sanger sequencing from both directions and incorporated into cells using PEI transfection and selection with G418 (InvivoGen, San Diego, CA, USA, ant-gn-5).

### 2.12. Confocal Microscopy

Approximately 200,000 cells with PCK2-ALFA expression were plated in 6-well plates containing 2 glass cover slips (12 mm; Deckglaser) in DMEM medium with 10% FBS. The following day, they were stained with 100 nM MitoTracker Deep Red (Invitrogen, Waltham, MA, USA) for 30 min before washing and formalin fixation. Cells were then stained with a 1:3000 dilution of FluoTag^®^-X2 anti-ALFA conjugated to Atto-488 (NanoTag Biotechnologies, Göttingen, Germany; N1502-At488) per the manufacturer’s directions. Cells were mounted to glass slides using Fluoromeount G DAPI (SouthernBiotech, Birmingham, AL, USA; 0100-20) and imaged on a Zeiss 800 CLSM with Airyscan at the UNMC Advanced Microscopy Core Facility.

### 2.13. Statistical Analysis

*p* values were calculated using Prism Software (GraphPad, v8.4.2, La Jolla, CA, USA). A *p* value of less than 0.05 was considered statistically significant. The statistical significance of these results was evaluated using one way ANOVA with multiple comparisons to knockdown in each cell line. The cell metabolic capacity assays were statistically evaluated using an unpaired, two-sided t-test to compare the effects of PCK2 depletion to control cells. Data are shown as mean +/– standard deviation (SD) unless otherwise noted.

## 3. Results

### 3.1. Endogenous PGC-1β Interacts with ERRα, Promotes ERRα Expression, and Anchorage-Independent Growth

**Figure 1:**
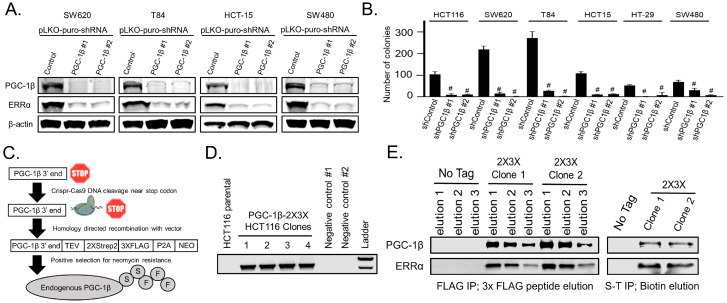
Endogenous PGC-1β interacts with ERRα, promotes ERRα expression, and increases anchorage-independent growth. (**A**) Lentiviral mediated transduction was used to express shRNAs for either a non-targeting control or two independent sequences targeting PGC-1β. The cells were lysed after 72 h and immunoblotted for PkfisGC-1β, ERRα, and loading control β-actin. (**B**) Colony formation in soft agar was measured after two weeks in cells infected with the same shRNAs from panel A. (**C**) Illustrative representation of the strategy used to knock in twin Strep2 and triple FLAG epitope tags onto the C-terminus of endogenous PGC-1β using CRIRSP-Cas9 mediated genome cutting with homology directed repair and neomycin selection. (**D**) Agarose gel showing PCR verification of correct genomic insertion of the epitope tags from part C in four clones of HCT116 cells. (**E**) HCT116 cells with epitope tagged endogenous PGC-1β were immunoprecipitated for either the FLAG or the Strep2 epitope and eluted with either the 3X-FLAG peptide or 50 mM biotin. Eluates were immunoblotted for PGC-1β and ERRα. # = *p*-value less than 0.001 when compared to the non-targeting control using one way ANOVA with multiple comparisons. S-T IP = Strep-Tactin immunoprecipitation.

We have previously shown that shRNA-mediated depletion of PGC-1β caused ERRα protein levels to decrease in human CRC cell line, HCT116 [3]. Here, we determined how robust this observation is by using lentiviral-mediated delivery of shRNAs to decrease PGC-1β expression in a panel of human CRC cell lines and immunoblotted for PGC-1β, ERRα, and measured anchorage-independent growth by colony formation in soft agar. Depletion of PGC-1β caused a decrease in ERRα protein levels and anchorage independent growth in a panel of K-Ras mutant CRC cell lines (Figure 1A,B).

PGC-1 proteins are known to bind transcription factors, but the physical interaction between the PGC-1β and ERRα has not been explored in detail. To investigate the physical interaction between PGC-1β and ERRα, we generated a vector for epitope-tagging of endogenous PGC-1β using homology directed repair (HDR). Using CRISPR-Cas9, we generated a double stranded break adjacent to the stop codon of PGC-1β and used our plasmid as a template for HDR to eliminate the stop codon and incorporate twin Strep2 triple FLAG epitopes and a neomycin resistance cassette (Figure 1C). After neomycin selection, clones were screened by PCR to confirm the correct genomic insertion (Figure 1D). Endogenous PGC-1β was immunoprecipitated by its FLAG epitopes and eluted with the 3X-FLAG peptide or immunoprecipitated by the Strep2 epitopes and eluted with biotin. Immunoblotting of the eluates showed both PGC-1β and ERRα, confirming their interaction (Figure 1E). These findings suggest PGC-1β binds ERRα to promote ERRα protein levels.

### 3.2. PGC-1β Requires Its LRELL Motif at Amino Acids 343–347 to Interact with ERRα

**Figure 2:**
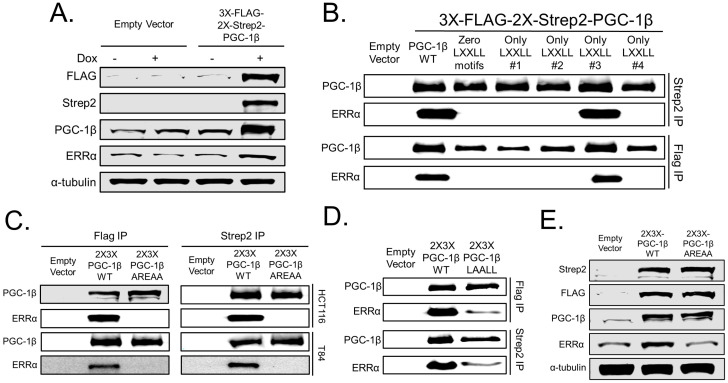
PGC-1β requires the LRELL motif at amino acids 343–347 to interact with ERRα. (**A**) HCT116 cells were cut at the AAVS1 safe harbor locus with CRISPR-Cas9 and homology-directed repair and puromycin selection were used to integrate an inducible full length PGC-1β cDNA with triple FLAG and twin Strep2 epitope tags on the N-terminus. Immunoblots for PGC-1β, Strep2, and FLAG confirm tagged protein induction, which causes increased ERRα expression. (**B**) HCT116 cells with either an empty vector, wild type dual epitope tagged PGC-1β, or five different PGC-1β mutants were immunoprecipitated for either the FLAG epitope or the Strep2 epitope and eluted with a triple FLAG peptide or 50 mM biotin, respectively. Eluates were subjected to immunoblot for PGC-1β and ERRα. (**C**) HCT116 and T84 cells with either an empty vector, wild type dual epitope tagged PGC-1β, or PGC-1β mutated at the LRELL motif to AREAA were immunoprecipitated as before and immunoblotted for PGC-1β and ERRα. (**D**) HCT116 cells with either an empty vector, wild type dual epitope tagged PGC-1β, or PGC-1β mutated at the LRELL motif to LAALL were immunoprecipitated as before and immunoblotted for PGC-1β and ERRα. (**E**) HCT116 cells expressing either wild type or the AREAA mutant PGC-1β from the AAVS1 safe harbor locus were treated with doxycycline for 24 h and lysed for Western blot to assess ERRα induction.

PGC-1 family proteins have been shown to use LxxLL amino acid motifs to bind transcription factors [9,10,11,12,13]. To determine the motif(s) required by PGC-1β to bind ERRα, we developed cell lines with inducible expression of N-terminus twin Strep2 triple FLAG epitope-tagged PGC-1β under doxycycline inducible expression from the AAVS1 safe harbor locus and found the increased levels of PGC-1β also causes a modest induction of ERRα protein levels (Figure 2A). We then made a series of PGC-1β mutant proteins to assess the role of each LxxLL motif in binding ERRα. To be in accordance with the previous labeling from the literature [14] we maintained the same labeling system: Motif 1 LLAEL (amino acids 92–96), Motif 2 LKQLL (amino acids 156–160), Motif 3 LRELL (amino acids 343–347), and Motif 4 LLSHL (amino acids 664–668). Technically, motifs 1 and 4 are reversed but we wanted to directly assess their role in ERRα binding since there is literature evidence to suggest these motifs may be functional in other PGC-1 family members [15]. Motifs were inactivated by mutating all leucines to alanines (LxxLL → AxxAA or LLxxL → AAxxA). Using this strategy, we created one quadruple PGC-1β mutant where all four motifs were inactivated (zero LxxLL motifs) and four triple mutants where only one LxxLL motif was left non-mutated (Only LxxLL motif #1, Only LxxLL motif #2, Only LxxLL motif #3, and Only LxxLL motif #4). The five mutant and wild type PGC-1β cDNAs were integrated into the AAVS1 safe harbor locus of HCT116 cells and the expressed proteins were immunoprecipitated using the FLAG or Strep2 epitopes in separate experiments. The eluates were immunoblotted for PGC-1β and ERRα and showed that the mutant PGC-1β that was functional at only the LxxLL motif #3 (LRELL) immunoprecipitated the same amount of ERRα as wild type PGC-1β (Figure 2B). To test the role of the LRELL motif, we made two additional mutants that had all three leucines mutated to alanines (LRELL → AREAA mutant) or the RE mutated to alanines (LRELL → LAALL mutant). The PGC-1β AREAA mutant cDNA was integrated into both HCT116 and T84 cells and tested against wild type PGC-1β. Loss of the leucines in motif #3 eliminated ERRα binding in both cell lines (Figure 2C). To test the role of amino acids RE in motif #3 on ERRα binding we generated a PGC-1β LAALL mutant cDNA that was integrated into HCT116 cells and tested against wild type PGC-1β. Mutation of amino acids RE showed only a partial loss of ERRα binding (Figure 2D). Lastly, we tested if the PGC-1β mutant that cannot bind ERRα could induce ERRα expression and found that the PGC-1β AREAA mutant was unable to induce ERRα expression compared to wild type PGC-1β (Figure 2E). Overall, these findings suggest that all five amino acids of the PGC-1β LRELL motif are required for optimal ERRα protein binding, which caused increased levels of ERRα protein.

### 3.3. PGC-1β and ERRα Promote PCK2 Expression

**Figure 3:**
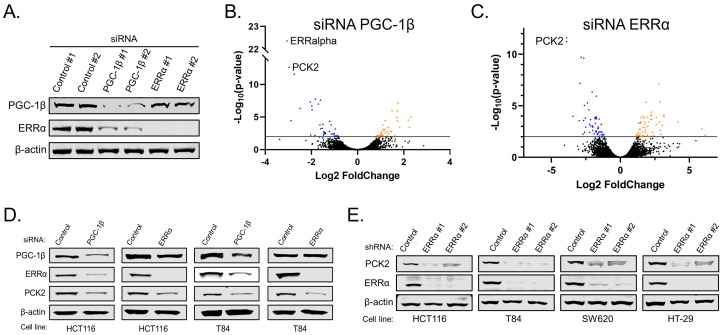
PGC-1β and ERRα promote PCK2 expression. (**A**) HCT116 cells were transfected with siRNAs targeting either PGC-1β or ERRα or a non-targeting control. After 72 h, cells were lysed and immunoblotted to confirm the depletion of the target proteins. (**B**,**C**) Volcano plots of RNAsequncing data from total RNA analysis 72 h after knockdown of PGC-1β or ERRα compared to a non-targeting control. (**D**) Colorectal cancer cell lines were transfected with siRNAs targeting either PGC-1β or ERRα and after 72 h were immunoblotted for PCK2. (**E**) Colorectal cancer cell lines were transduced with lentiviruses expressing shRNAs targeting ERRα or a non-targeting control and after 96 h were lysed and immunoblotted for PCK2 and ERRα.

The genes regulated by PGC-1β and ERRα have only been examined in detail in breast cancer and normal liver tissue [16,17,18], but not in CRC. To determine which genes were regulated by PGC-1β and ERRα in CRC, we validated two siRNAs that targeted either protein (Figure 3A) and that loss of PGC-1β caused decreased levels of ERRα, but decreased levels of ERRα did not alter PGC-1β expression. To determine the genes regulated by PGC-1β and ERRα, we transfected HCT116 cells with siRNAs targeting either PGC-1β, ERRα, or non-targeting controls and collected total RNA after 72 h for RNA sequencing (Supplemental File S6). First, depletion of endogenous PGC-1β led to a dramatic decrease in ERRα mRNA, consistent with the model in the literature that binding of PGC-1 proteins to ERRα increases ERRα transcriptional activity by decreases inhibitory phosphorylation and that ERRα can bind to its own promoter to increase ERRα mRNA and protein levels (Figure 2E and 3B) [18,19,20,21]. Secondly, mitochondrial Phosphoenolpyruvate Carboxykinase 2 (PCK2) decreased the most after depletion of both PGC-1β and ERRα (Figure 3B,C). PCK2 is localized to the mitochondria and catalyzes the irreversible conversion of oxaloacetate (OAA) to phosphoenolpyruvate (PEP). Lastly, several other genes that regulate amino acid metabolism decreased after depletion of both PGC-1β and ERRα, suggesting that these two proteins cooperate to promote amino acid incorporation and metabolism to increase survival of colorectal cancer cells.

To confirm that the mRNA changes seen after depletion of PGC-1β and ERRα lead to changes in PCK2 protein expression we performed transient knockdown of either protein in two CRCcell lines and observed decreased levels of PCK2 by Western blot (Figure 3D). Lastly, we used lentiviral mediated delivery of shRNA targeting ERRα to reduce ERRα expression in three CRC cell lines and observed decreased levels of PCK2 by Western blot (Figure 3E). These findings suggest the transcriptional control of PCK2 levels by PGC-1β and ERRα is a mechanism to control amino acid metabolism in CRC.

### 3.4. PCK2 Promotes Anchorage-Independent Growth and Glutamine Utilization

**Figure 4:**
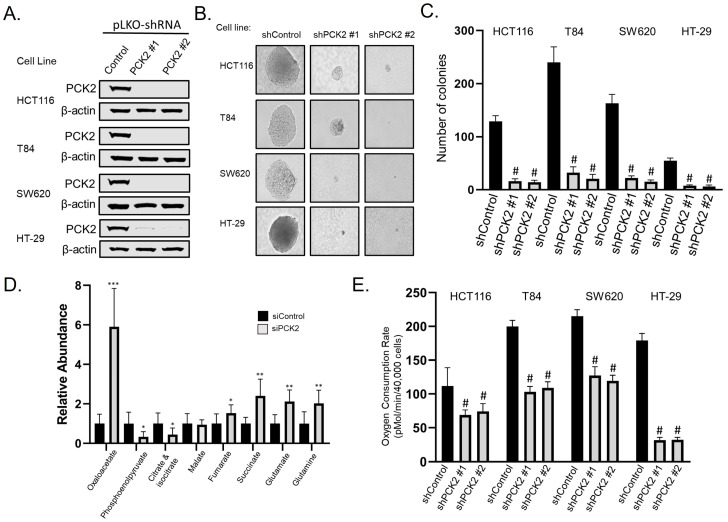
PCK2 promotes anchorage independent growth and glutamine utilization. (**A**) Colorectal cancer cell lines were transduced with lentivirus producing shRNAs targeting PCK2 and subjected to immunoblot after 72 h to confirm knockdown. (**B**) Representative photomicrographs of colonies in soft agar two weeks after shRNA mediated depletion of PCK2 in four CRC cell lines (**C**) Quantification of colonies from panel B. (**D**) Intracellular metabolites were measured by LC-MS/MS 72 h after siRNA-mediated depletion of PCK2. (**E**) Oxygen consumption rates were measured using a Seahorse XFe96 analyzer in colorectal cancer cell lines 72 h after shRNA-mediated knockdown of PCK2 using only L-glutamine as a substrate. * = *p*-value less than 0.05; ** = *p*-value less than 0.01, *** = *p*-value less than 0.001. # = *p*-value less than 0.001 when compared to the non-targeting control using one way ANOVA with multiple comparisons.

PCK2 mRNA has been shown to be upregulated by mutant K-Ras [22], but its functional role has not been explored in CRC. We have previously shown that PGC-1β expression is dependent on mutant K-Ras signaling [3], suggesting that transcriptional control of PCK2 by PGC-1β is part of oncogenic K-Ras mediated metabolic reprogramming. To assess the role of PCK2 in CRC survival we used lentiviral-mediated delivery of shRNAs targeting PCK2 to deplete PCK2 protein (Figure 4A) and performed anchorage-independent growth studies. We found that depletion of PCK2 caused a significant decrease in soft agar colony formation in a panel of K-Ras mutated CRC cell lines (Figure 4B,C).

To determine how depletion of PCK2 alters metabolism, we transiently depleted PCK2 in HCT116 cells and measured intracellular metabolites using liquid chromatography and tandem mass spectrometry (Figure 4D). PCK2 is responsible for the irreversible conversion of oxaloacetate (OAA) to phosphoenolpyruvate (PEP) within the mitochondria. As expected, depletion of PCK2 caused a six-fold increase in total oxaloacetate (OAA) levels and a 55% decrease in total phosphoenolpyruvate (PEP) levels. Unexpectedly, elevated levels of OAA did not appear to be converted into citrate and isocitrate, as the pool of citrate/isocitrate was lower in cells with PCK2 depletion. Depletion of PCK2 also caused an accumulation of upstream TCA metabolites fumarate, succinate, glutamate, and glutamine, suggesting that glutamine flux through the TCA cycle was diminished in the absence of PCK2 activity. To assess L-glutamine utilization, we used shRNAs to deplete PCK2 in a panel of CRC cell lines and measured their oxygen consumption rate using a Seahorse Metabolic Analyzer using only L-glutamine as a substrate. Depletion of PCK2 caused a significant decrease in L-glutamine oxidation in four K-Ras mutant CRC cell lines (Figure 4E). Overall, these findings suggest that PCK2 can control glutamine flux through the TCA cycle to promote CRC cell survival.

### 3.5. ERRα Depletion Causes Loss of Anchorage-Independent Growth and Glutamine Utilization That Is Not Rescued by Over-Expression of PCK2

**Figure 5:**
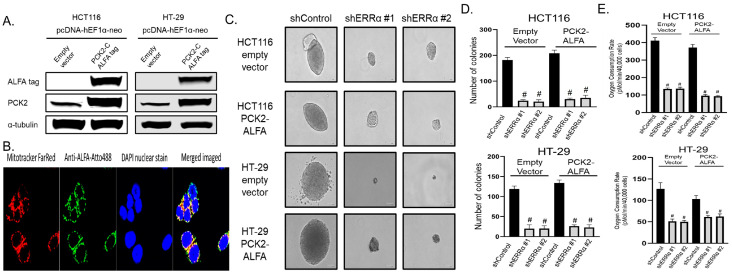
ERRα depletion causes loss of anchorage-independent growth and glutamine utilization and that is not rescued by elevated PCK2 levels. (**A**) Immunoblot of colorectal cancer cell lines showing stable expression of PCK2 with a C-terminus ALFA epitope tag after neomycin selection. (**B**) Confocal photomicrographs showing the overlap of PCK2 with mitochondrial dye. (**C**) Representative photomicrographs of colonies in soft agar two weeks after shRNA mediated depletion of ERRα in two CRC cell lines with plasmid derived PCK2 expression or empty vector control (**D**) Quantification of colonies from panel C. (**E**) Oxygen consumption rates were measured using a Seahorse XFe96 analyzer in CRC cell lines from panels C and D using only L-glutamine as a substrate. # = *p*-value less than 0.001 when compared to the non-targeting control using one way ANOVA with multiple comparisons.

To assess if PCK2 can rescue the loss of ERRα activity, we developed a novel plasmid for stable PCK2 expression. PCK2 is anchored to the mitochondrial membrane at its N-terminus so we added an ALFA epitope tag [23] to the C-terminus and expressed it via a human EF-1α promoter in two CRC cell lines using neomycin selection (Figure 5A). We confirmed that the PCK2-ALFA protein was successfully localized to the mitochondria by performing direct immunofluorescence for the ALFA epitope using an ALFA epitope recognizing nanobody conjugated to Atto-488 and compared it to MitoTracker Far Red staining (Figure 5B).

To determine if over-expression of PCK2 could rescue the loss of ERRα, we used lentiviral mediated delivery of shRNAs targeting ERRα in two cell lines stably expressing PCK2-ALFA or an empty vector and performed anchorage-independent growth and L-glutamine utilization assays. Loss of ERRα caused a significant loss of colony formation and L-glutamine metabolism that was not rescued by the over-expression of PCK2 (Figure 5C–E). These findings suggest that transcriptional control of PCK2 expression is one mechanism used PGC-1β and ERRα to promote glutamine metabolism and CRC cell survival, but that other PGC-1β and ERRα target genes are also important to CRC metabolism and survival.

## 4. Discussion

Here, we show that PGC-1β and ERRα physically interact and promote genes that increase amino acid metabolism. PGC-1β has been shown to regulate several metabolic processes in other model systems [2,19,24,25,26], but the regulation of PCK2 and amino acid metabolism is a novel observation in CRC that differs from previous studies. Our study is the first to examine the role of PCK2 in CRC metabolism and survival and our observations suggest that PCK2 maximizes flux through the TCA cycle by converting OAA to PEP to promote cell survival. Our finding that elevated levels of OAA after siRNA-mediated depletion of PCK2 were not converted to citrate and isocitrate suggests that there are insufficient levels of either citrate synthetase or its other substrate Acetyl-CoA. Using Western blot, we were able to easily detect citrate synthetase in all CRC cell lines tested. We did not measure levels of Acetyl-CoA in the mitochondria. These data are consistent with reports that the mitochondrial pyruvate complex is down-regulated in colorectal cancer [27,28], which would limit the import of pyruvate into the mitochondria for conversion into Acetyl-CoA. These findings suggest intramitochondrial levels of Acetyl-CoA are insufficient to convert excess OAA to citrate and that conversion of OAA to PEP, presumably for mitochondrial export, is the most efficient method for utilizing L-glutamine by the TCA cycle in CRC.

Our data show that PGC-1β uses all five amino acids of its LRELL motif to bind ERRα protein, but no other LxxLL or LLxxL motif appears to bind ERRα, which suggests to us that PGC-1β can only bind one ERRα molecule at a time. ERRα has been shown to directly bind DNA at Estrogen-Related Receptor Response Elements (ERRE) (TNAAGGTCA) to increase transcription [18,29]. However, ERRα has over 800,000 predicted binding sites in the human genome, raising the possibility that additional transcription factors are required for gene selection by PGC-1β. Additionally, mapping of the ERREs shows several near the PCK2 core promoter, but many are greater than several kilobases from the transcriptional start site (TSS) suggesting again that additional factors are required to bring the PGC-1β/ERRα complex to the TSS of target genes to increase transcription. Here, we have created several novel PGC-1β mutant proteins that can be used to assess the role of each LxxLL and LLxxL motif alone or in combination to determine their role in transcription factor binding and to discover new components of PGC-1β signaling. Additionally, PGC-1 family proteins have been shown to bind Host Cell Factor proteins through a DHDY motif [30], which represents another potential mechanism that PGC-1β may use for gene selection. Host Cell Factor 1 and 2 have been proposed to bridge transcriptional co-activators, such as PGC-1 family proteins, to DNA binding via transcription factor binding at their N-terminus Kelch repeat domains, but this process has not been fully explored.

Our study has clinical implications for patients with K-Ras mutations. In patients with liver metastases of CRC, K-Ras mutations have been shown to be a negative prognostic marker of overall survival [31,32,33,34]. We have previously shown that PGC-1β expression is dependent on mutant K-Ras [3,4] and that targeting the PGC-1β signaling pathway would represent a novel target for precision medicine in tumors with K-Ras mutations. Currently, there are no treatments that directly inhibit PGC-1β. Our data suggest that targeting ERRα or PCK2 could act as a treatment strategy in K-Ras mutant tumors. Several groups have developed inverse agonists that bind the ERRα ligand binding pocket and force it into an inactive conformation [35,36,37,38,39,40,41]. Our data from (Figure 2E and 3B) show that the binding of PGC-1β to ERRα dramatically increases ERRα activity and expression and suggest that preventing the interaction between PGC-1β and ERRα would inhibit ERRα signaling through an alternate mechanism to inhibitors of the ligand binding pocket. Defining the domain on PGC-1β that is required to bind ERRα represents the first step toward developing inhibitors that would block the PGC-1β/ERRα interaction. Similar efforts are underway to identify selective inhibitors of PCK2, but current inhibitors also target PCK1 with minimal selectivity between the two paralogs [42,43,44,45]. Inhibition of either ERRα or PCK2 would represent a novel treatment strategy that should be tested in pre-clinical models of CRC.

Lastly, our study is limited in the following ways: (1) All the experiments were performed in the human CRC cell lines with K-Ras mutations. Although K-Ras mutated tumors represent approximately 40% of all CRC, additional testing is required to see if our results translate to tumors with either wild type K-Ras, mutant BRAF, or HER-2/neu over-expression. (2) We identified several genes regulated by both PGC-1β and ERRα, but only focused on the role of PCK2 in glutamine metabolism. Over-expression of PCK2 was unable to rescue the loss of ERRα, suggesting that additional PGC-1β/ERRα target genes are required for K-Ras induced metabolic change. Further studies to define the role of additional PGC-1β/ERRα target genes will further elucidate the scope of these metabolic changes. (3) Our metabolite analysis of CRC cells with and without PCK2 was limited to whole cell extracts and measured by LC-MS/MS. However, metabolite concentrations can vary across different subcellular compartments. For example, the depletion of PCK2 caused decreased levels of total levels of PEP, which consists of the PEP generated by PCK2 in the mitochondria combined with PEP from other sources. Advancements in methodology will be needed to more precisely define how metabolites change within the mitochondria after the depletion of PCK2. (4) PGC-1α is the PGC-1β paralog with the most amino acid similarity to PGC-1β and has been more intensively studied. The literature has shown that PGC-1α and ERRα directly interact, and multiple reports have characterized the interacting alpha helices by solving the crystal structure of this critical interaction [11,12,13]. Based on the literature, we assume that PGC-1β and ERRα directly interact, but we did not directly test this. (5) We did not directly map the binding of ERRα to the PCK2 gene as we felt this would be best interpreted in the context of a more complex understanding of the PGC-1β/Host Cell Factor proteins interaction.

## 5. Conclusions

Inhibiting amino acid metabolism in CRC with K-Ras mutations by targeting PGC-1β signaling has the potential to provide cancer cell specific therapy without subjecting the patient to the stresses of global metabolic restriction.

## Data Availability

Raw sequence data has been deposited as GSE147905 in the National Center for Biotechnology Information Gene Expression Omnibus. Scripts used to perform these analyses can be found at https://github.com/j-berg/frodyma_2020 (accessed on 31 March 2020).

## Supplementary Materials

The following supporting information can be downloaded at: https://www.mdpi.com/article/10.3390/cancers14194879/s1, Supplemental File S1: shRNA sequences; Supplemental File S2: PGC-1B homology arms; Supplemental File S3: Mutagenesis primers; Supplemental File S4: siRNA sequences; Supplemental File S5: Raw images for Western blot figures; Supplemental File S6: RNAseq differential expression list.
