# Supplementary material for: PGC-1β and ERRα Promote Glutamine Metabolism and Colorectal Cancer Survival via Transcriptional Upregulation of PCK2
Source: Cancers (Basel). 2022 Oct 5;14(19):4879. doi: 10.3390/cancers14194879 (PMC9562873; doi:10.3390/cancers14194879)
Supplement: Supplementary file 1 [file cancers-14-04879-s001.zip › cancers-1920605-supplementary.pdf]

Supplemental File S1

pLKO-shRNA-puro sequences

PGC-1β #1	5’CGAGCTCTCACTGCTGCAGAA
PGC-1β #2	5’GGCGGACAGCACCCAAGACAA
ERRα #1	5’GCAGAGCAATAACACTATATT
ERRα #2	5’CCCTGCAGAGCAATAACACTA
PCK2 #1	5’GTAGAGAGCAAGACGGTGATT
PCK2 #2	5’GCACATCCCAACTCTCGATTT

Supplemental File S2

5’ homology arm with 2X Strep tag and TEV site (HOM 1)

TCCCCGACCTGCAGCCCAGCTCCCAGCACTTTGGGAGGCCGAGGCAGGAAGATCATGAG
GTCAAGAGATCGAGACCATCGTGGCCAACATGGTGAAACCCCCTCTCTATTAAAAATACAA
AAATTAGCTGGGCGTGGTGGCGCACACCTGTAGTCCCAGCAACTTGGGAGGCTGAGGCG
GGAGAATCACTTGAACCCGAGAGGCAGAGGTTGCAGTGAGCTGAGATCACATCCCTGCAC
TCCAGCCTTGGTGACAGAGCGAGACTGCATCTCAAAAAAAAATAAATTCTAGTGTTGTTAG
CCCTCCCTTATGTGGCACTGCAGCAGGTTACTAATGGACGAGAAGCTGTTGGGGGAAGTA
GAGTTGTAGGGTGTTGGAGCTAGAAAGGCTCTGGAGTGCTCTAGTTTGGGCCTCCAGGTC
TCTAGATAGGACAGCCAAGGCCCTGAGACACTGATACCATGGCCAAGATGTCCCAGCAGG
ATGGCAGGCAGTGCCCACAGTCCAGCGCTCATCCCAGCTCCCCAAGGCCTTGGGCTGCA
GCCACTCCACGGTGCCCACTCATAGTGGCAGATTCTTCAACCATCCGGTCTTTGTAAGTTG
CTCACTGCCTTCCCCTCTTCCCTGCCTCTTCACCCCCATGCCCAGATTCCAATTCAGAAGA
GGCCCTTCCTGCGTCAGGGAAAAGCAAGTATGAAGCCATGGATTTTGACAGCTTACTGAAA
GAGGCCCAGCAGAGCCTGCATGAGAACCTGTACTTCCAATCCAATGGGAGCGGAGGAGG
TTCCGG

3’ homology arm (HOM 2)

AGTTCTTCTGATTCGAACATCAGACAAGGCCCTTCCAATATGTTTACGTTTTCAAAGAAATC
AAGTATATGAGGAGAGCGAGCGAGCGTGAGAGAACACCCGTGAGAGAGACTTGAAACTGC
TGTCCTTTAAAAAAAAAAAAAATCAATGTTTACATTGAACAAAGCTGCTTCTGTCTGTGAGTT
TCCATGGTGTTGACGTTCCACTGCCACATTAGTGTCCTCGCTTCCAACGGGTTGTCCCGGG
TGCACCTCGAAGTGCCGGGTCCGTCACCCATCGCCCCTTCCTTCCCGACTGACTTCCTCT
CGTAGACTTGCAGCTGTGTTCACCATAACATTTCTTGTCTGTAGTGTGTGATGATGAAATTG
TTACTTGTGAATAGAATCAGGACTATAAACTTCATTTTTAATTGAAAAAAAAAGTATATCCTT
AAAATAATGTATTTATGGCTCAGATGTACTGTGCCTGGGATTATTGTATTGCTTCCTTGATTT
TTTAACTATGCACTGTCATGAGGTGTTTGCCACTGAGCTGCCCTGCTCCCCTTGCCAGATT
GCCCTGGAGGTGCTGGGTGGCCGCTAGGCTGGTCTGCAGGAAAGCGCGGCCTGCCGTTT
CCGGGCCGTATCTGCCAAGCCCTGCCTTGTCTCTTACTGAGCAAGTTTGGCTCAAATTATA
GGAGCCCCCATCTTGTGCCCAGCTCATGCTCCAAGTGTGTGTCTATCCATTTGTACTCAGA
CTCTTGAGTACCTTGTAAGGAAGGCGGGGCAAGCTCTTGGAAAGTCCTCTCCA

Cas 9 gRNA sequence to cut PGC-1β adjacent to the stop codon:

5’ GATAACAGCCTTAACCCTCG

PCR primers for verification of genomic insertion

Forward primer 5’ CAGGACTGAGTGTCAGTGACC

Reverse primer 5’ CAGCAGGCTGAAGTTAGTAGC

Supplemental File S3

LLxxL 1 into AAxxA

```
          E   N   E   A   A   A   A   E   A   T   K   T   L
LA1-F 5'GT GAG AAT GAG GCC GCC GCT GCA GAG GCC ACC AAG ACC CTG G
LA1-R 5'C CAG GGT CTT GGT GGC CTC TGC AGC GGC GGC CTC ATT CTC AG
```

LxxLL 2 into AxxAA

```
         E   L   S   L   A   Q   K   A   A   L   A   T   S   Y
LA2-F 5'C GAG CTC TCA CTG GCT CAG AAG GCC GCC CTG GCC ACA TCC TAC
LA2-R 5'GTA GGA TGT GGC CAG GGC GGC CTT CTG AGC CAG TGA GAG CTC G
```

LxxLL 3 into AxxAA

```
        E   F   S   I   A   R   E   A   A   A   Q   D   V   L
LA3-F 5'GAG TTC TCC ATT GCC AGG GAA GCT GCT GCT CAA GAC GTG CTC
LA3-R 5'GAG CAC GTC TTG AGC AGC AGC TTC CCT GGC AAT GGA GAA CTC
```

LLxxL 4 into AAxxA

```
          E   R   S   E   A   A   S   H   A   R   H   A   T
LA4-F 5'CA GAG CGA AGT GAG GCC GCT TCC CAC GCC CGA CAT GCC ACA G
LA4-R 5'C TGT GGC ATG TCG GGC GTG GGA AGC GGC CTC ACT TCG CTC TG
```

LRELL into LAALL (RAEA)

```
            E   F   S   I   L   A   A   L   L   A   Q   D   V
RAEA-F 5'CT GAG TTC TCC ATT CTG GCC GCC CTT CTG GCT CAA GAC GTG
RAEA-R 5'CAC GTC TTG AGC CAG AAG GGC GGC CAG AAT GGA GAA CTC AG
```

Supplemental File S4

siRNA sequences and product number

Target	Item #	Target Sequence
PGC1β	J-008556-06	CCAGAAGGCGUCCUGCAAA
PGC1β	D-008556-02	GUACAGAACUACAUAAGCA
ERRα	J-003403-07	GGCCUUCGCUGAGGACUUA
ERRα	J-003403-08	GCGAGAGGAGUAUGUUCUA
PCK2	J-006797-07	GAGCAAGACGGUGAUUGUA
PCK2	J-006797-09	CCUGGGAGAUGGUGACUUU
Non-Targeting 1	D-001810-01	UGGUUUACAUGUCGACUAA
Non-Targeting 2	D-001810-02	UGGUUUACAUGUUGUGUGA

Supplemental File S5 - Original Labeled Images for Figures S1-S5

Figure S1A – SW620 panels

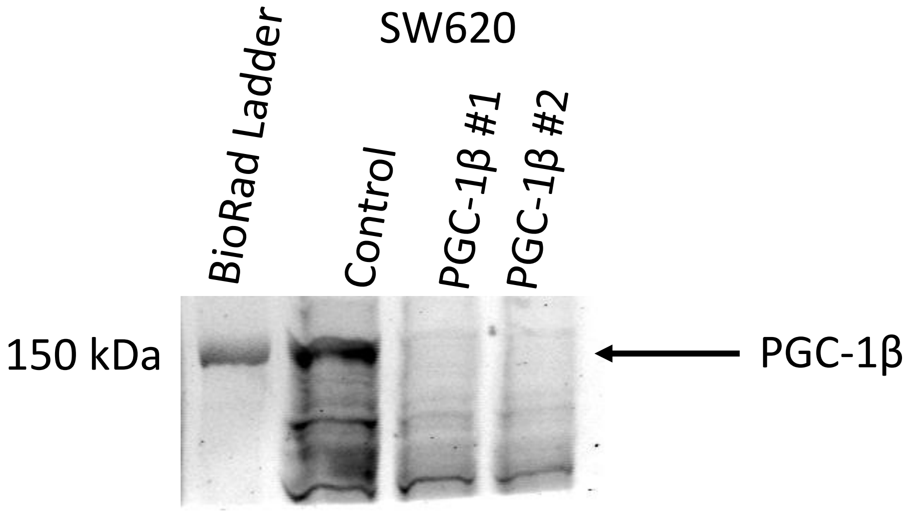

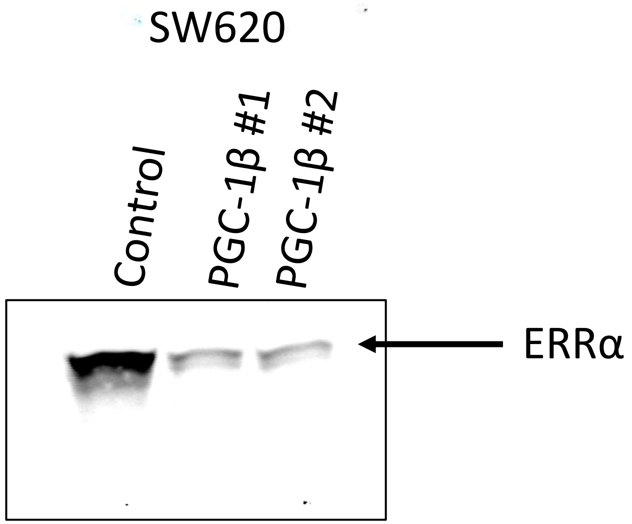

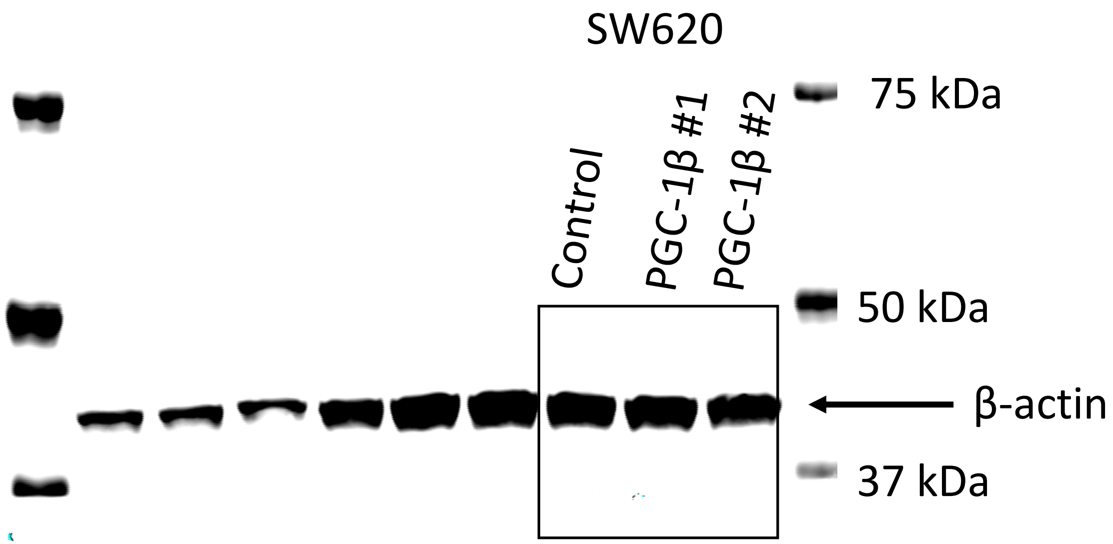

Figure S1A Continued – T84 panels

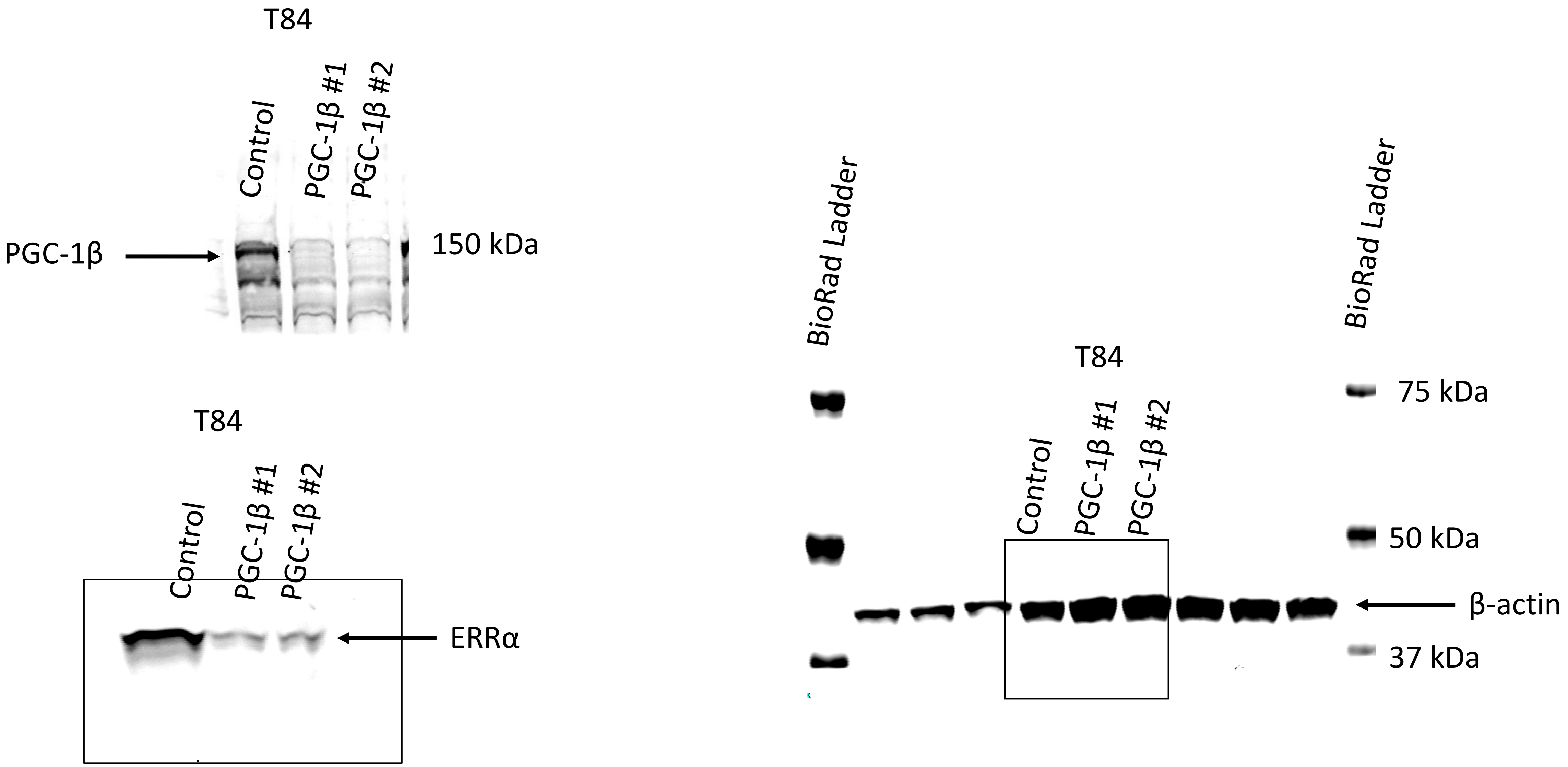

Figure S1A Continued – HCT-15 panels

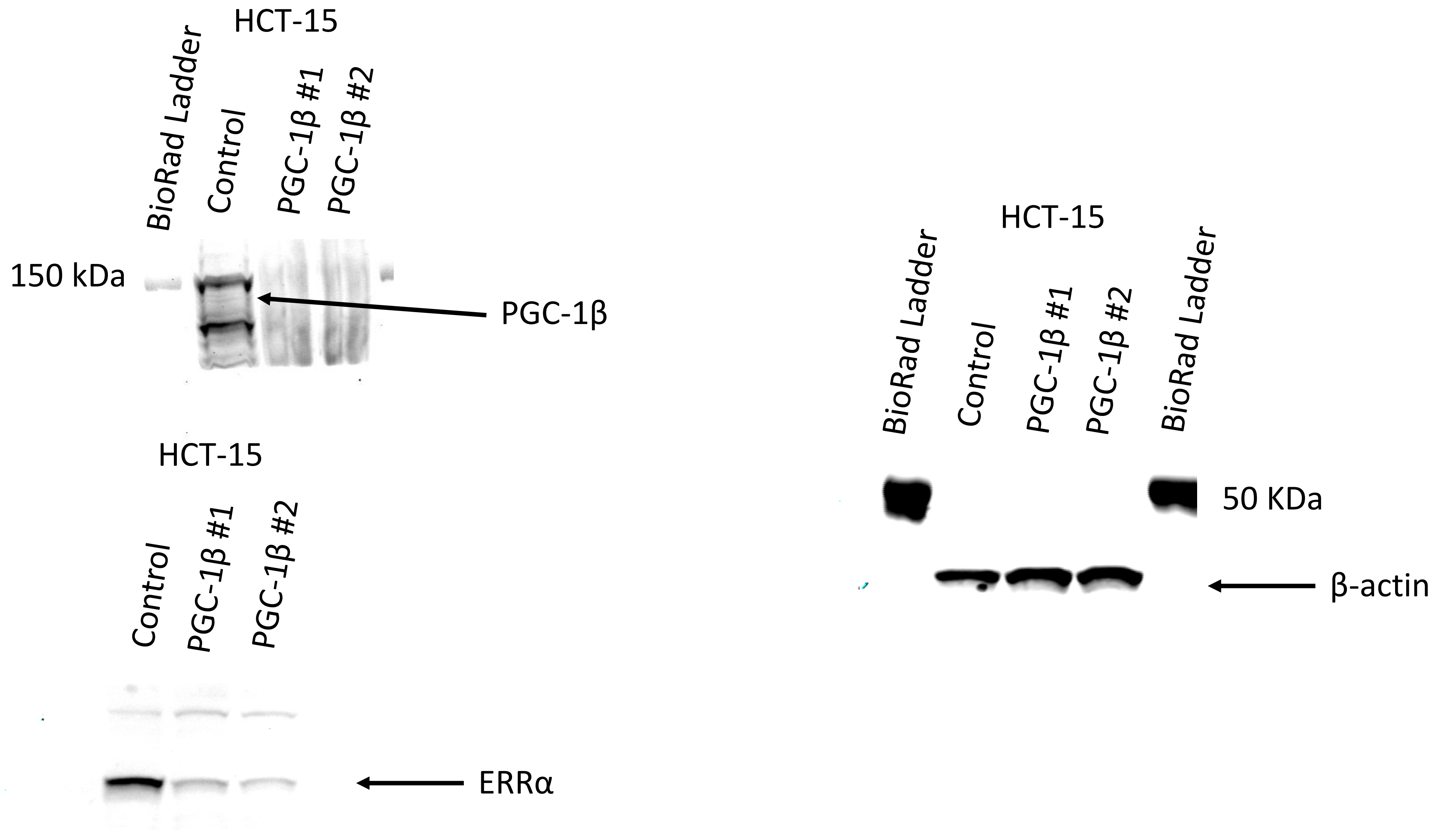

Figure S1A Continued – SW480 panels

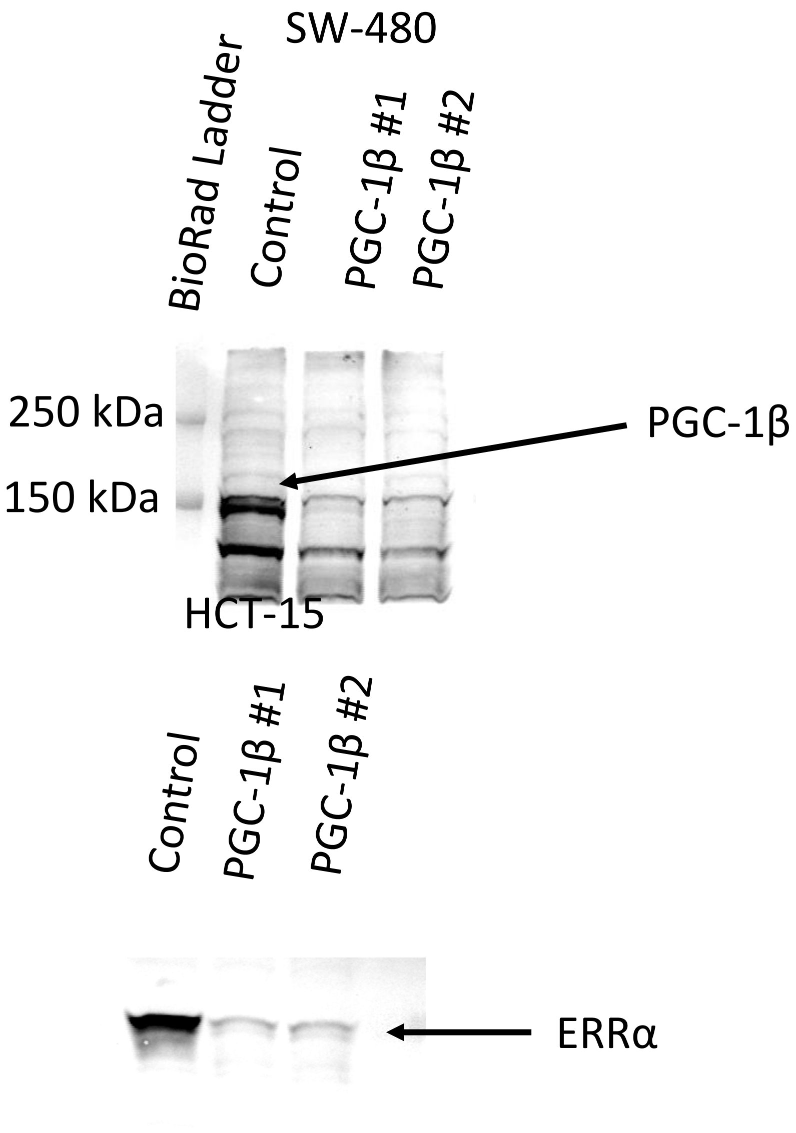

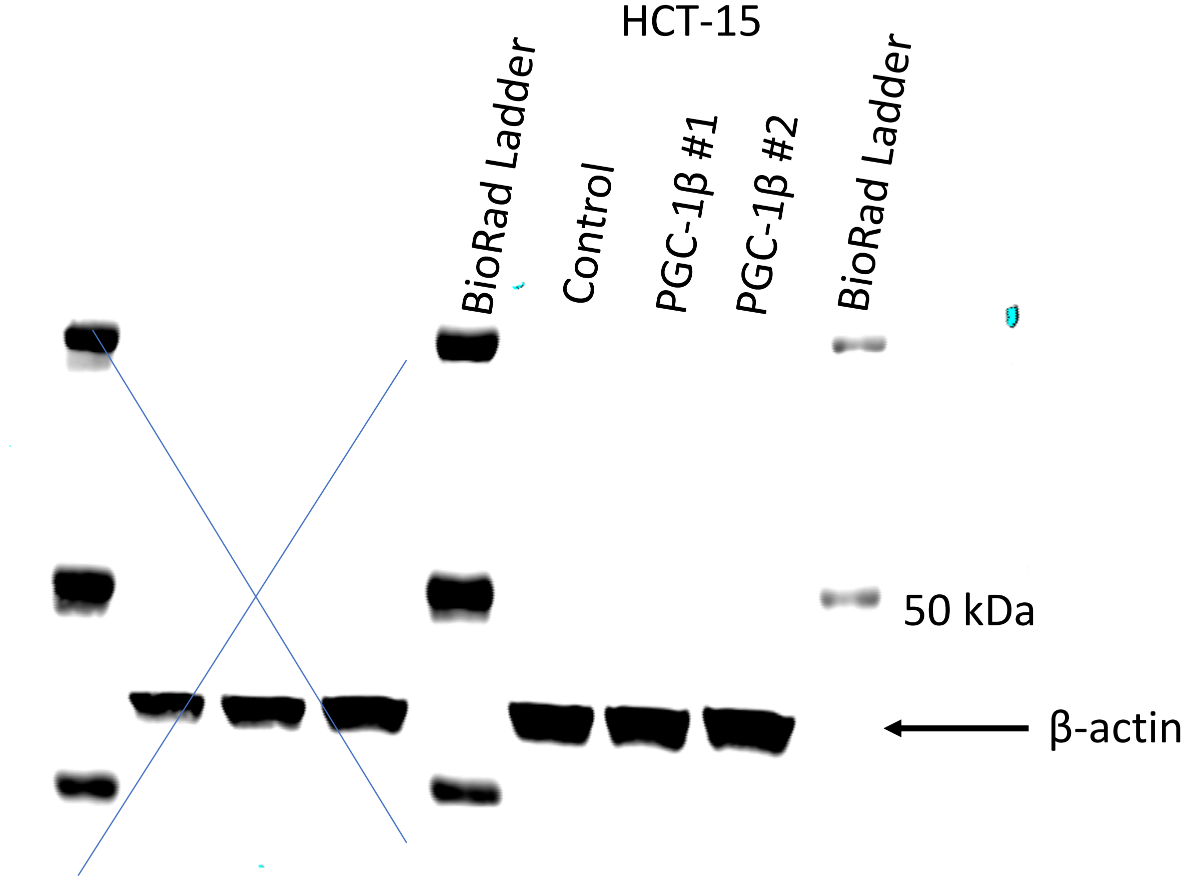

Figure S1D – Agarose Gel

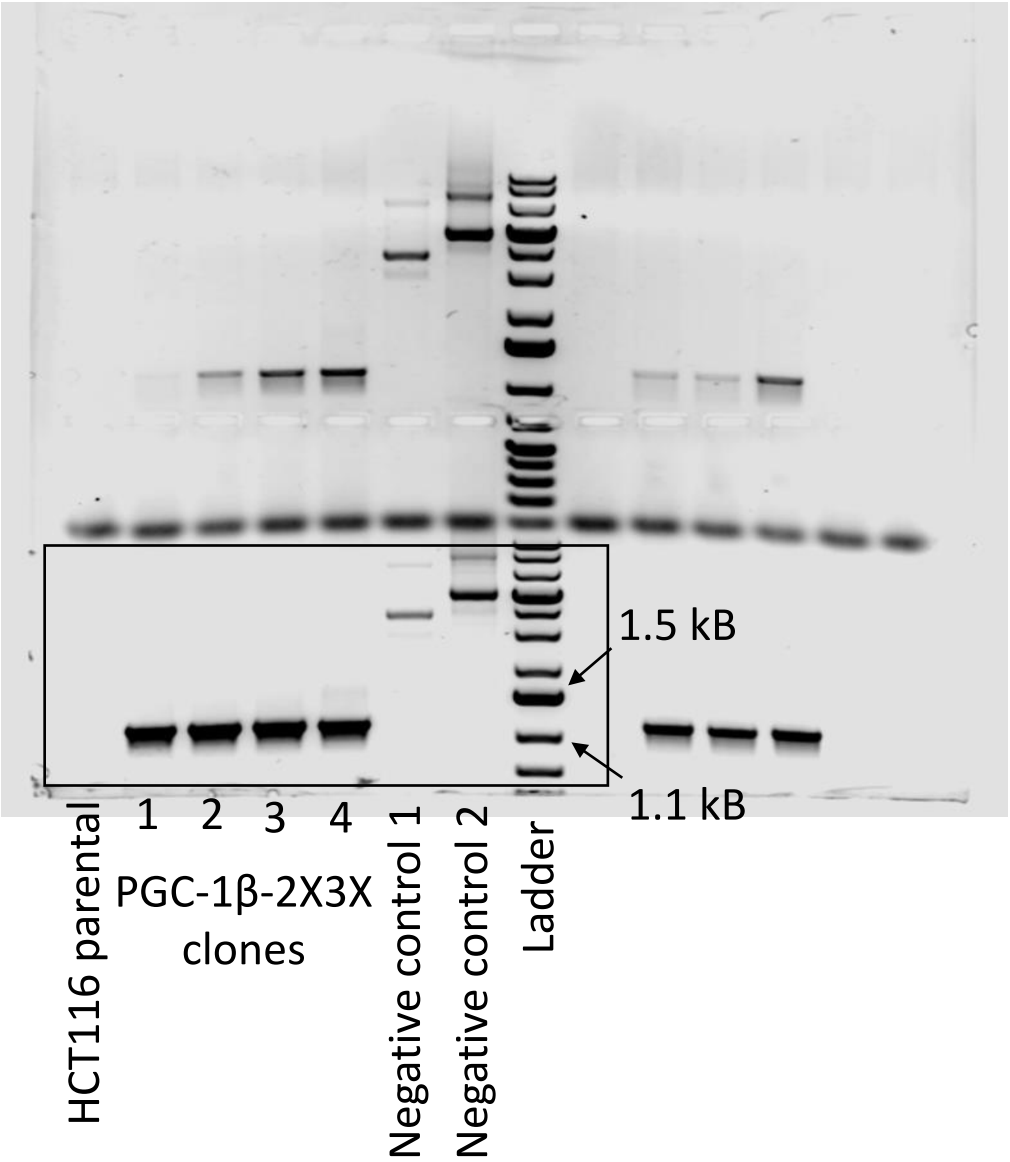

Negative control 1 –
Vector pFETCH

Negative control 2 –
HDR vector

Figure S1E – Western Blot, FLAG IP

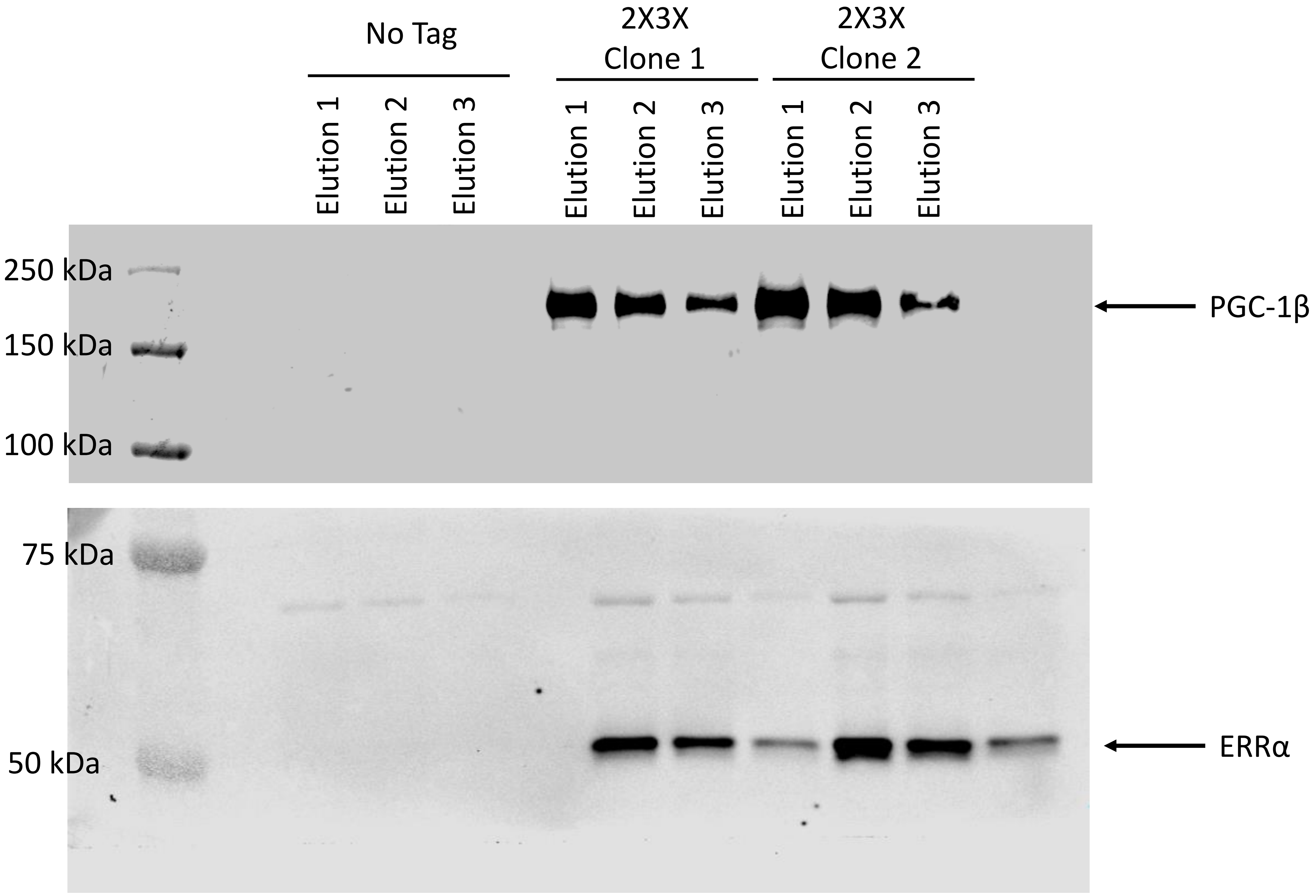

Figure S1E – Western Blot, Strep-Tactin IP

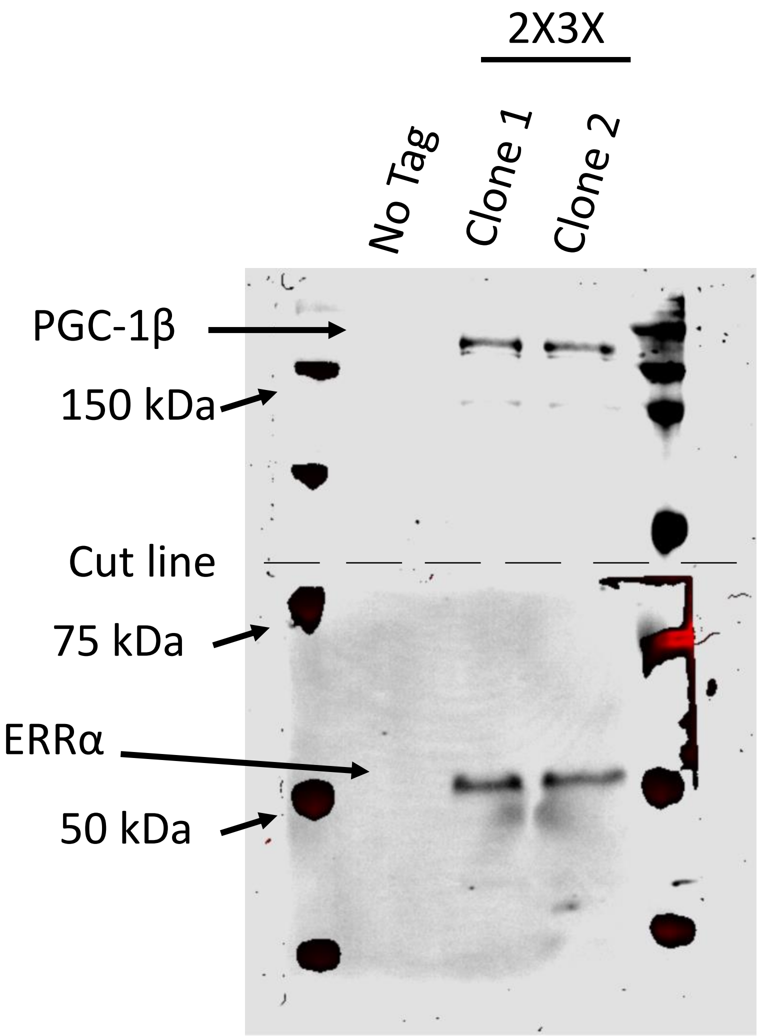

One blot, cut in half, top half probed for PGC-1β, and bottom half probed for ERRα

Figure S2A

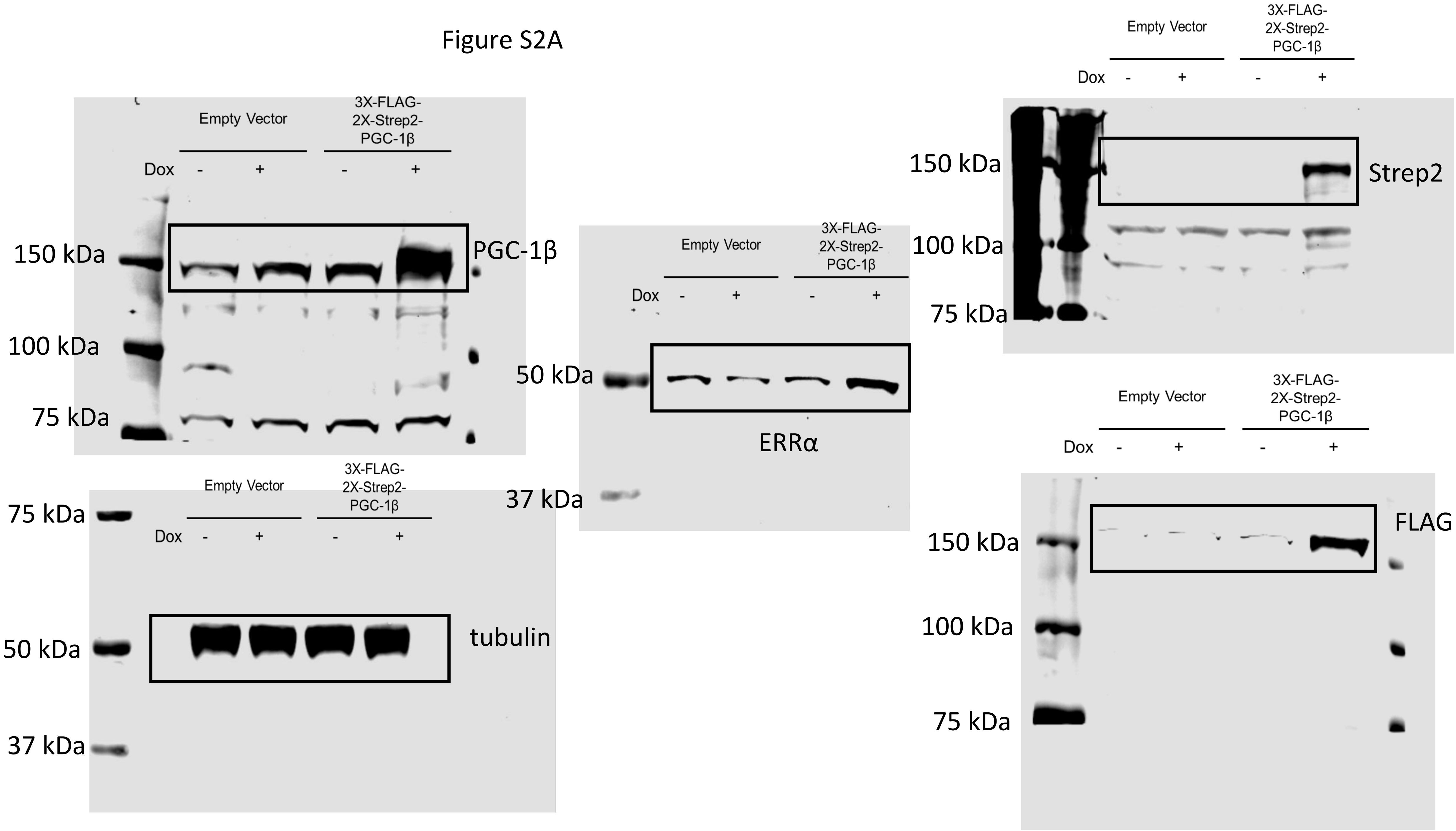

Empty Vector
3X-FLAG-
2X-Strep2-
PGC-1β
Dox - + - +
150 kDa
100 kDa
75 kDa
PGC-1β
50 kDa
37 kDa
ERRα
tubulin
Strep2
FLAG

Figure S2B –

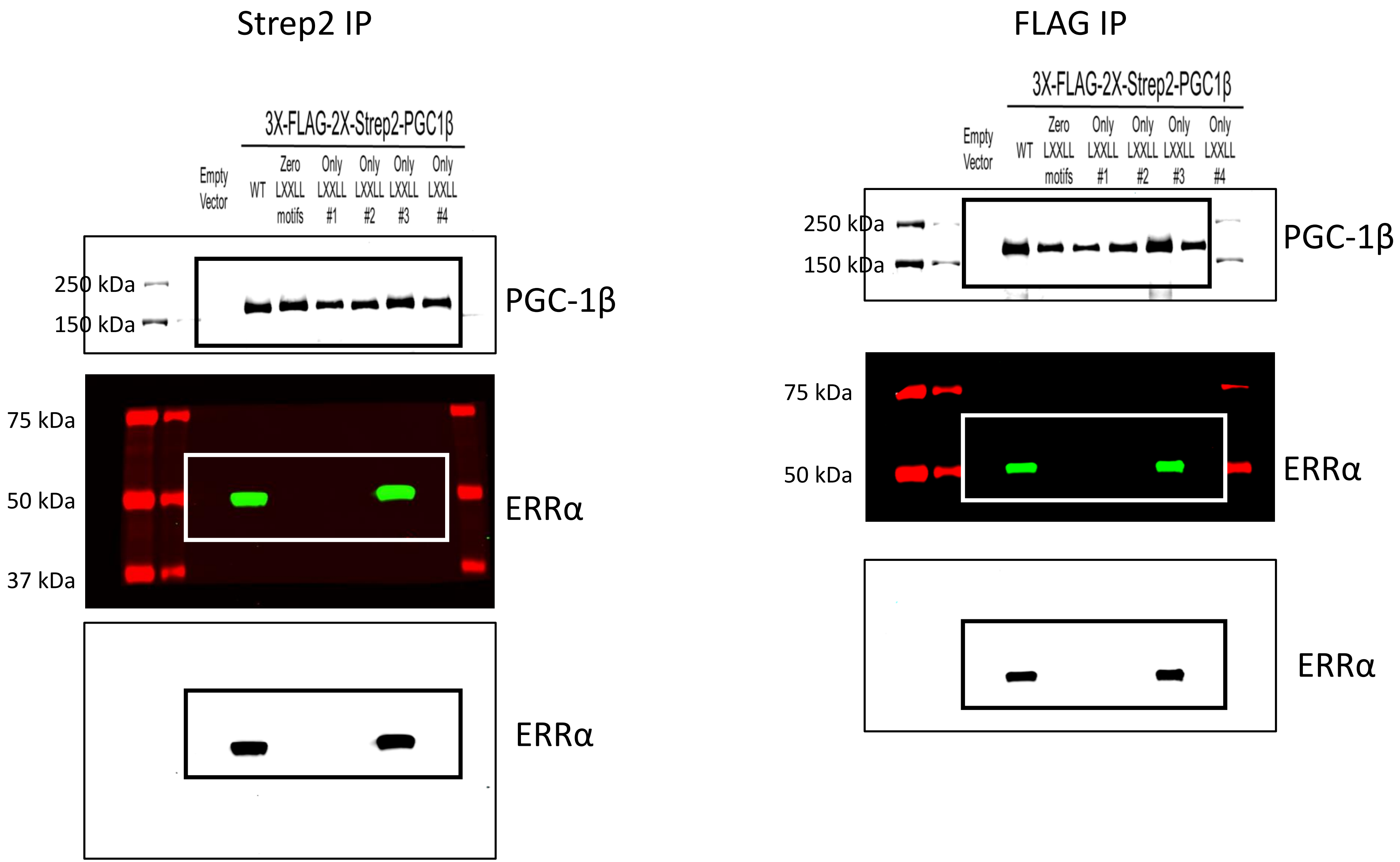

Strep2 IP
3X-FLAG-2X-Strep2-PGC1β
Empty Vector
WT
Zero LXXLL motifs
Only LXXLL #1
Only LXXLL #2
Only LXXLL #3
Only LXXLL #4
250 kDa
150 kDa
PGC-1β
75 kDa
50 kDa
37 kDa
ERRα
ERRα
FLAG IP
3X-FLAG-2X-Strep2-PGC1β
Empty Vector
WT
Zero LXXLL motifs
Only LXXLL #1
Only LXXLL #2
Only LXXLL #3
Only LXXLL #4
250 kDa
150 kDa
PGC-1β
75 kDa
50 kDa
ERRα
ERRα

Figure S2C – HCT116 cell line

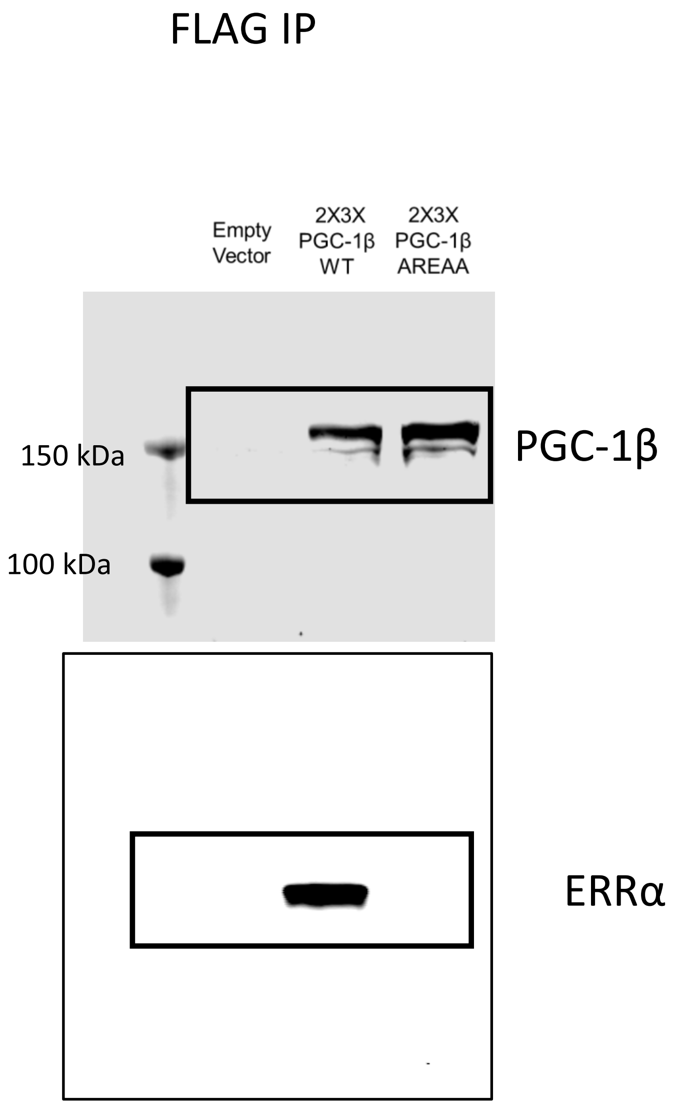

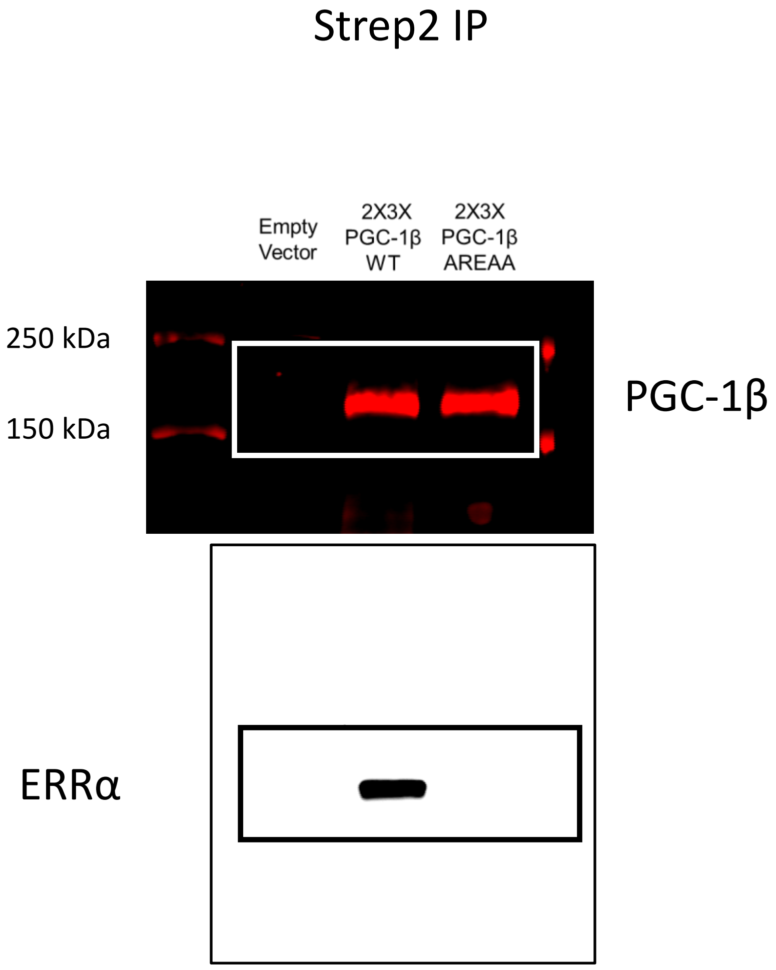

Figure S2C – T84 cell line

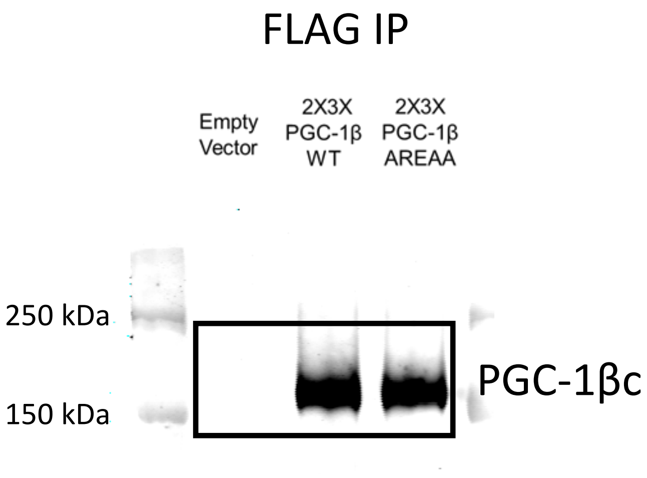

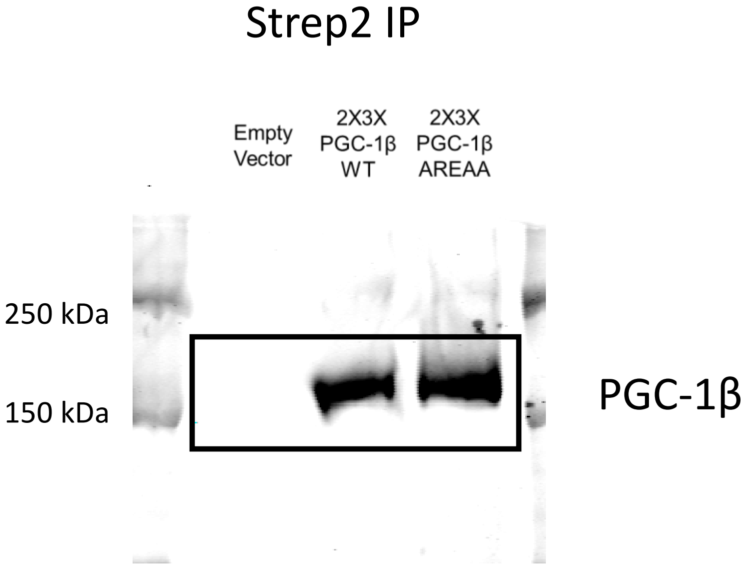

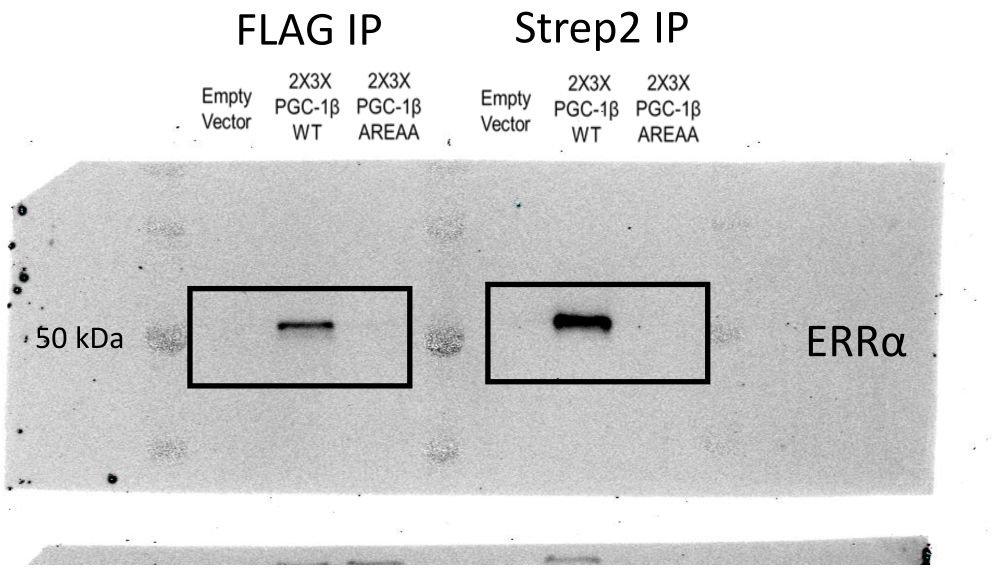

Figure S2D –

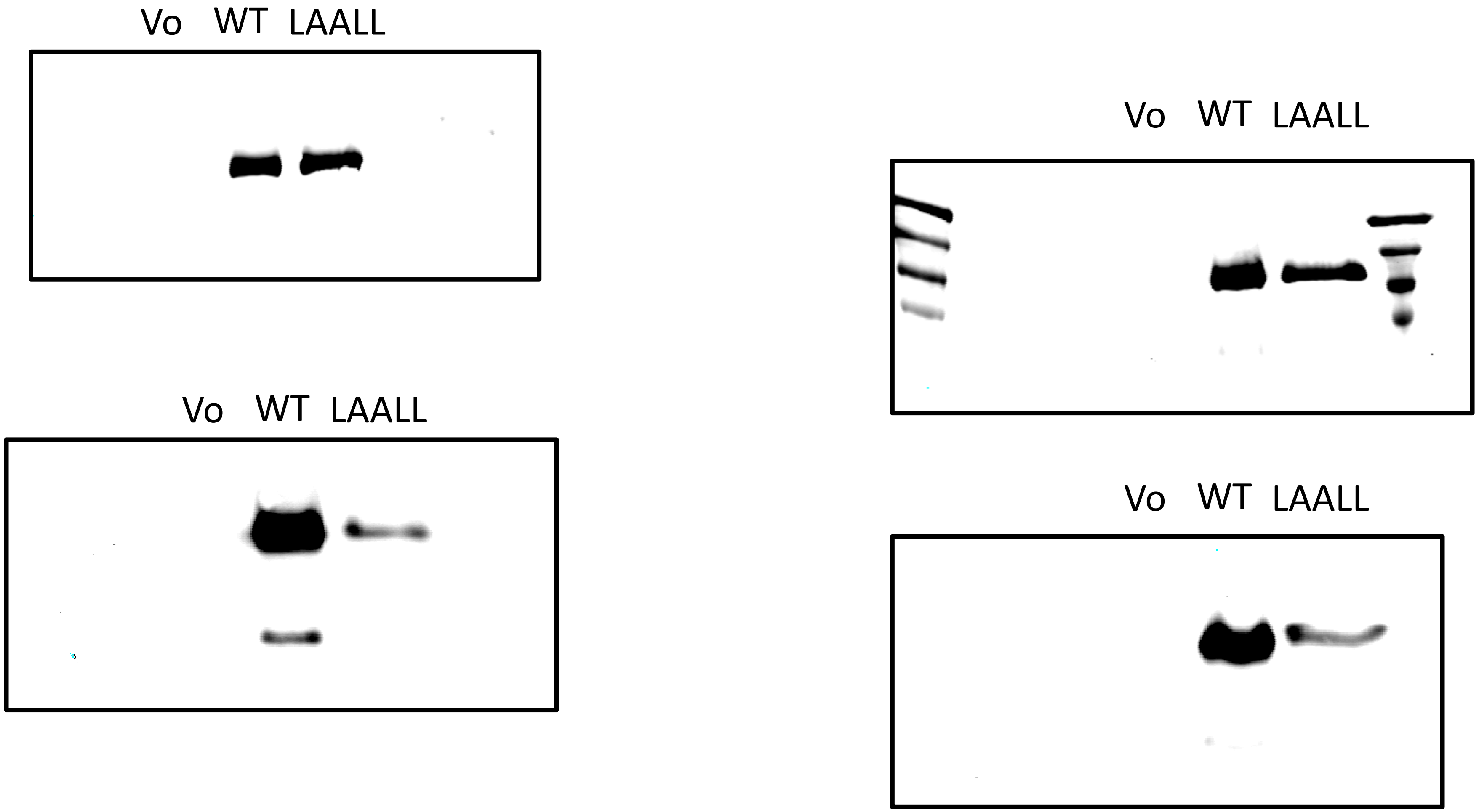

Figure S2E –

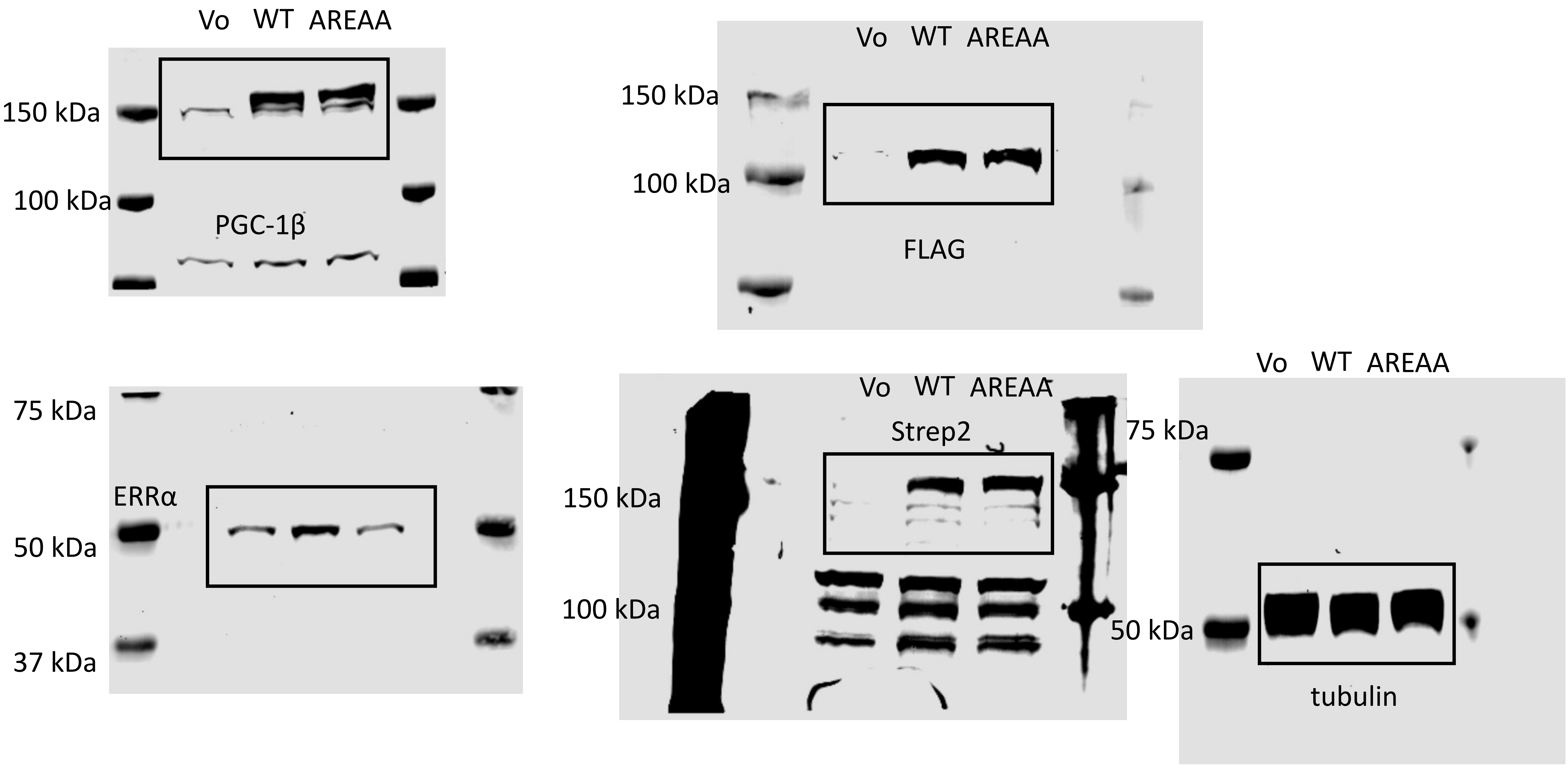

Vo WT AREAA
150 kDa
100 kDa
PGC-1β
Vo WT AREAA
150 kDa
100 kDa
FLAG
75 kDa
ERRα
50 kDa
37 kDa
Vo WT AREAA
Strep2
150 kDa
100 kDa
Vo WT AREAA
75 kDa
50 kDa
tubulin

Figure S3 A

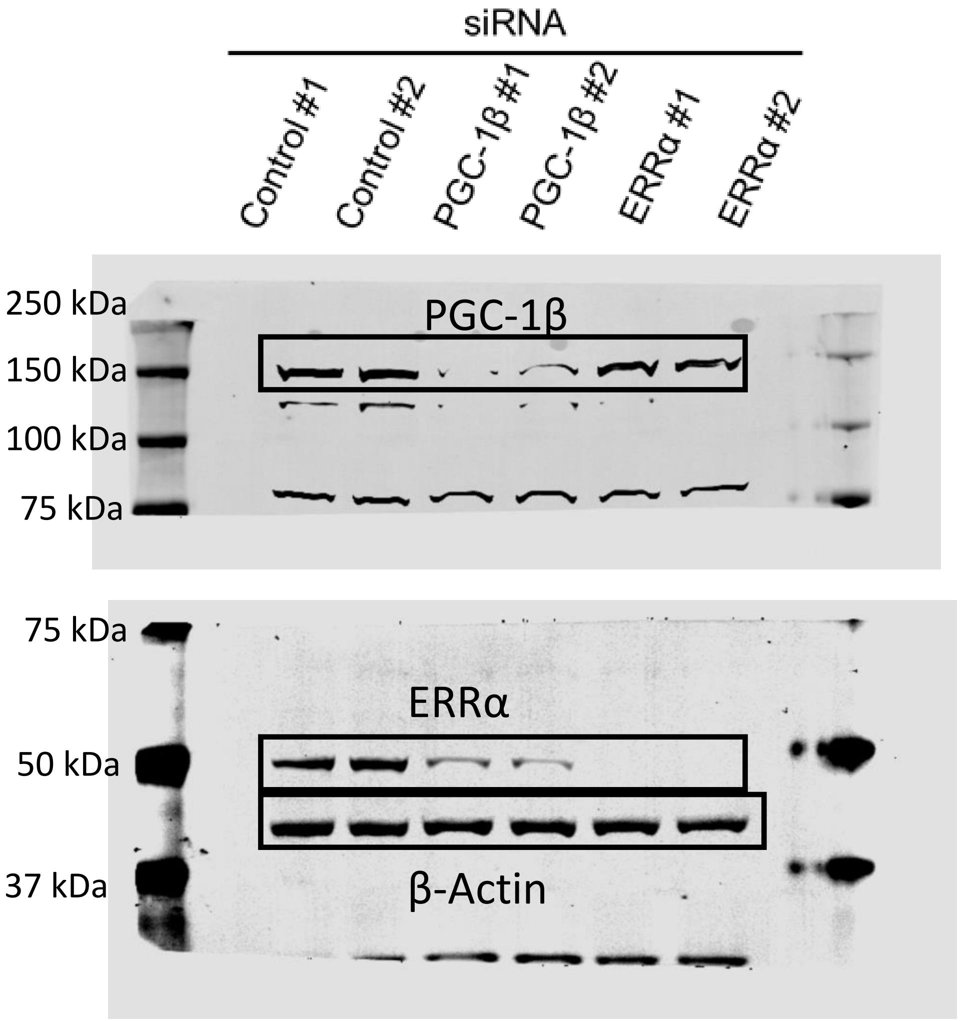
siRNA
Control #1
Control #2
PGC-1β #1
PGC-1β #2
ERRα #1
ERRα #2
250 kDa
150 kDa
100 kDa
75 kDa
PGC-1β
75 kDa
50 kDa
37 kDa
ERRα
β-Actin

Figure S3D – HCT116 siPGC-1Beta

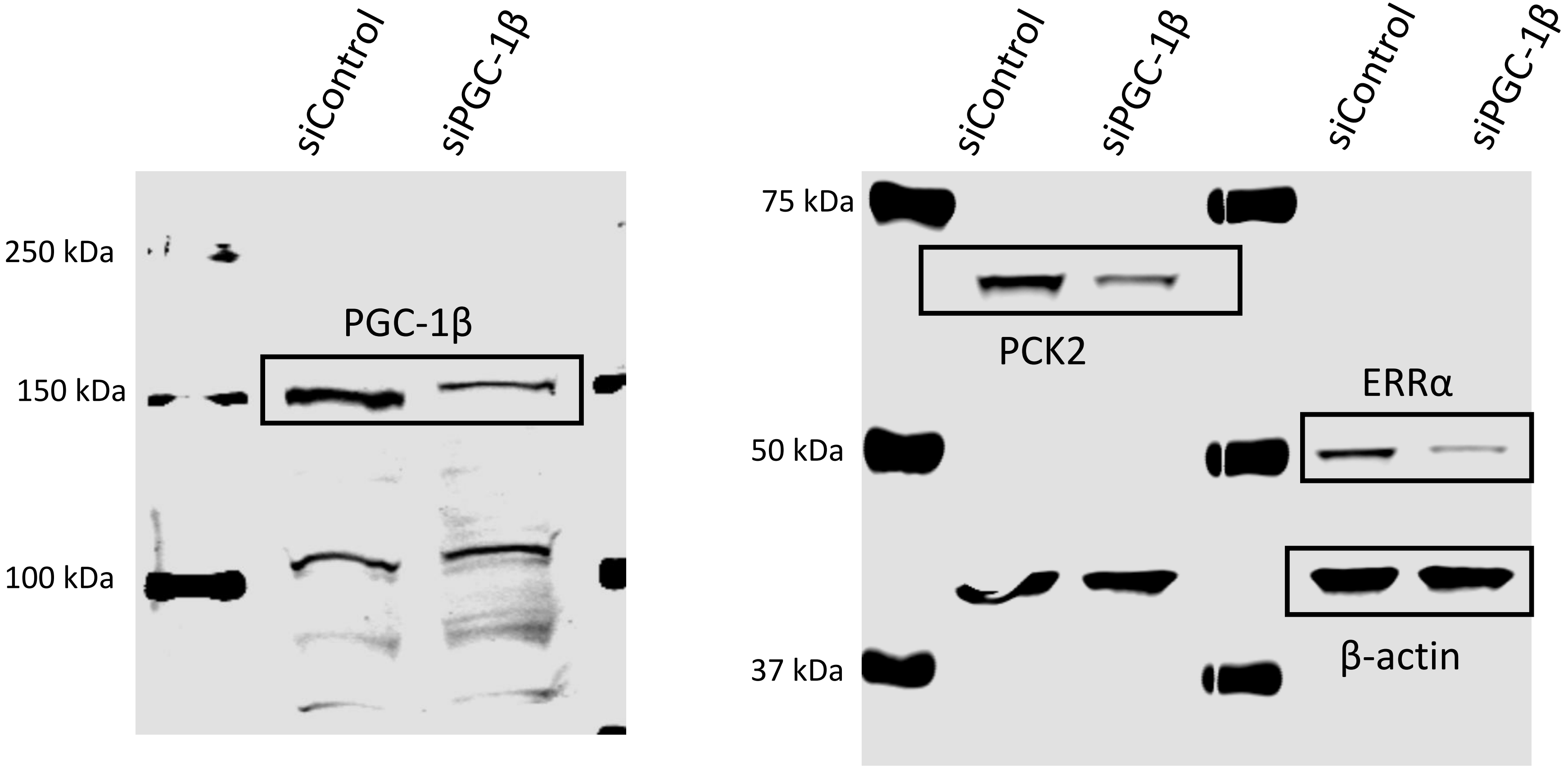

Figure S3D – HCT116 siERRalpha

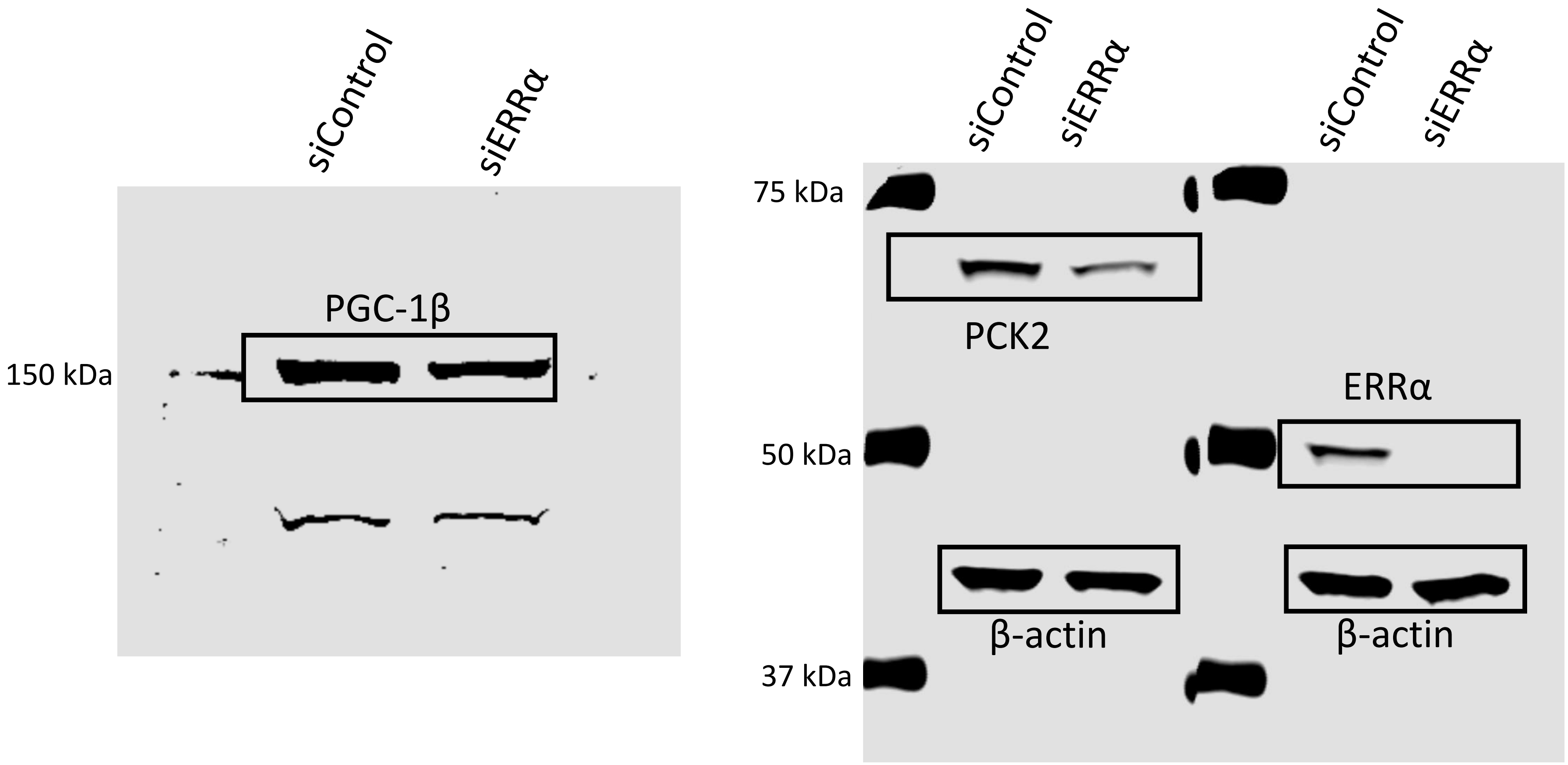

Figure S3D –T84 siPGC-1Beta

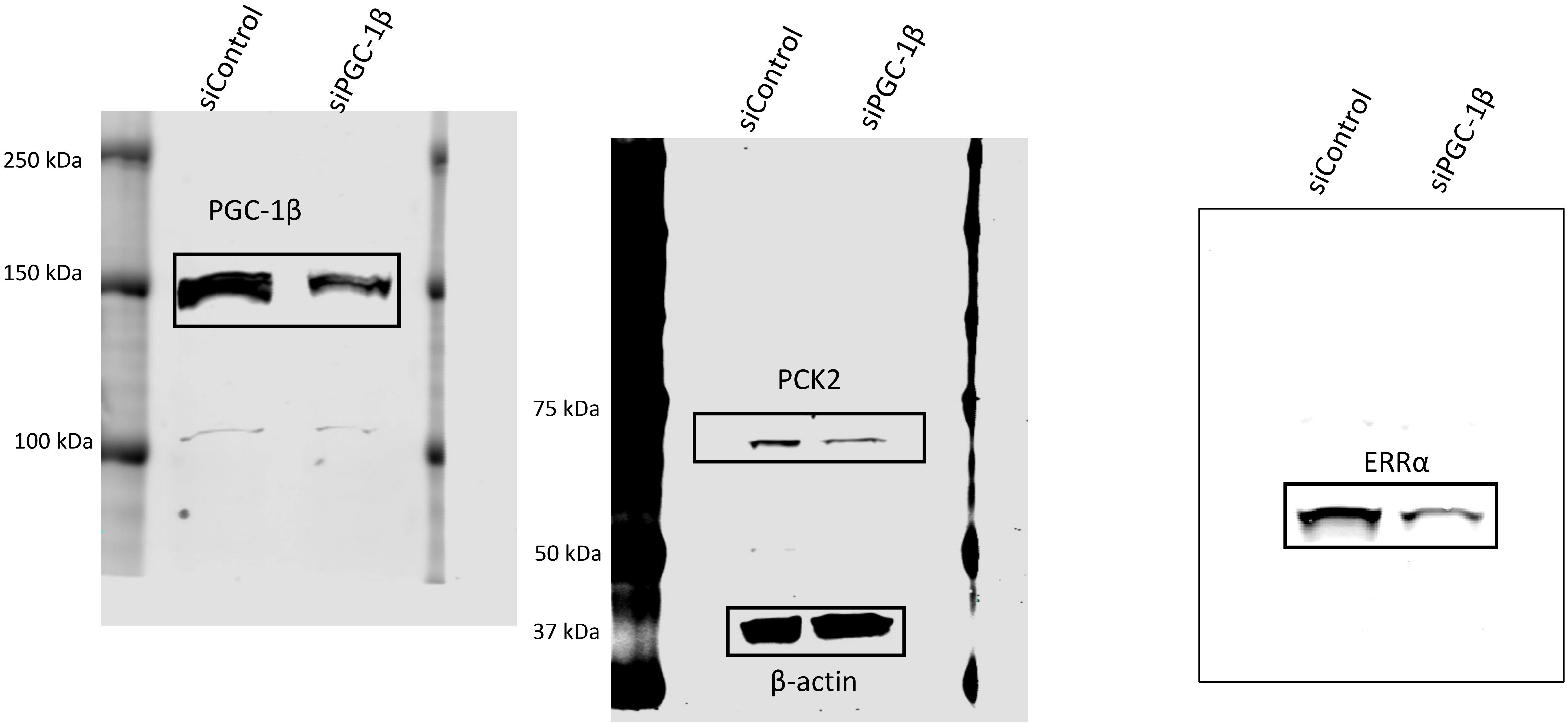

Figure S3D – T84 siERRalpha

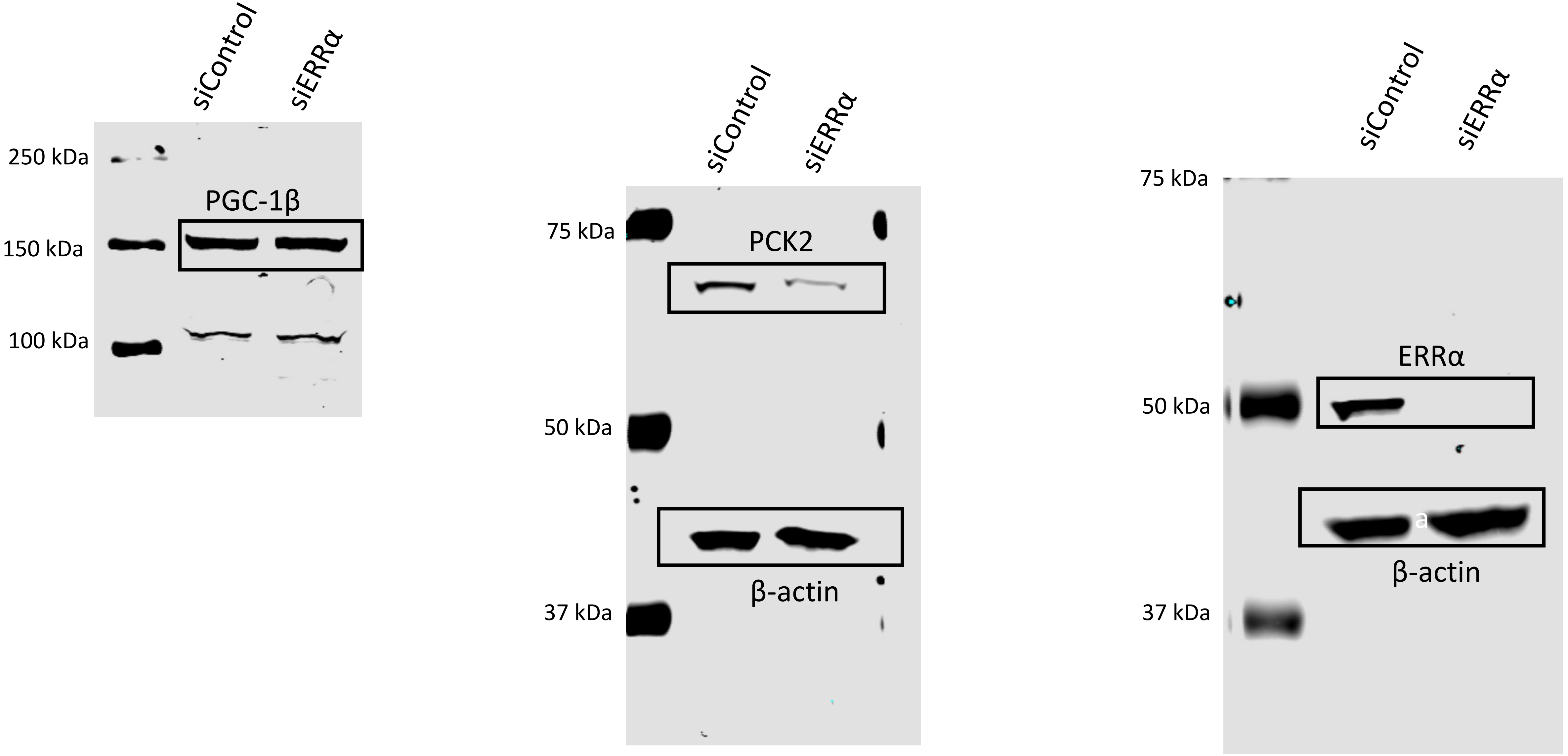

Figure S3E – shERRa to PCK2 WB

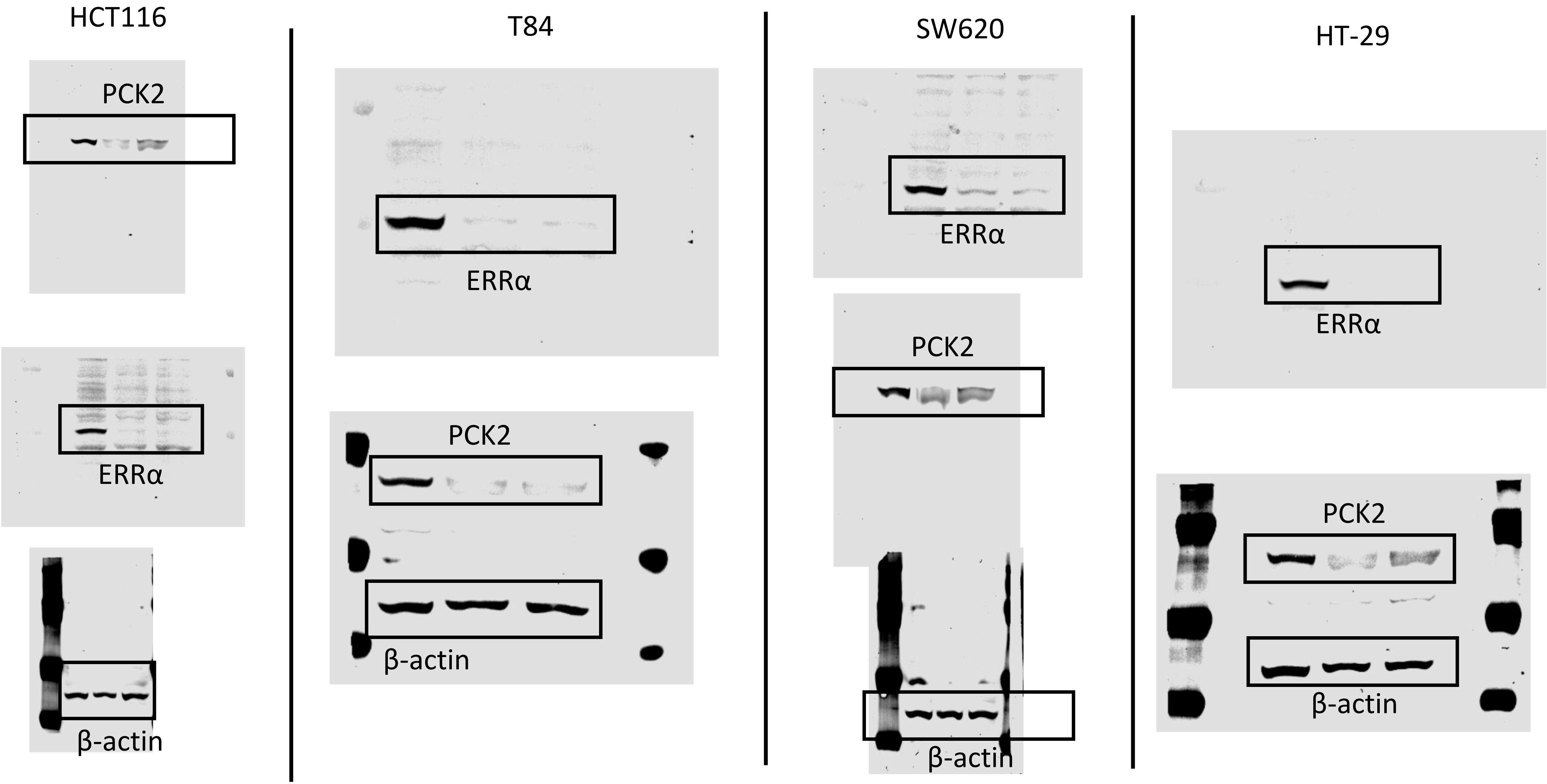

Figure S4A – All four cell lines

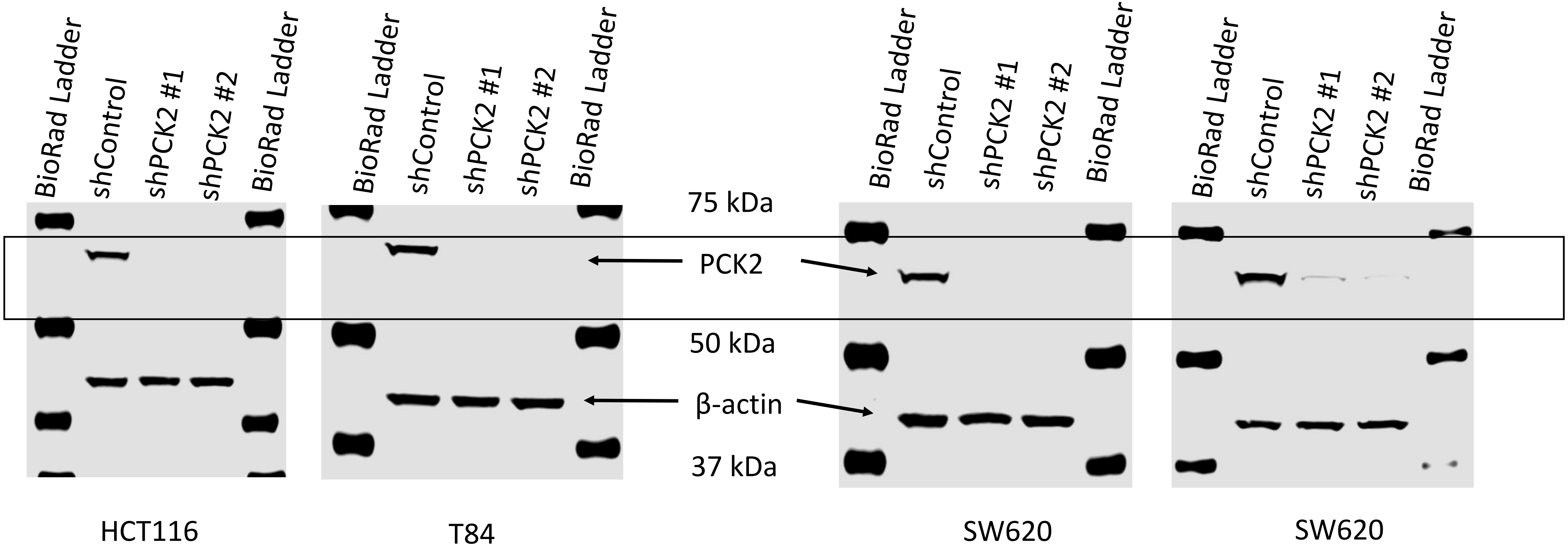

Figure S5A – HCT116

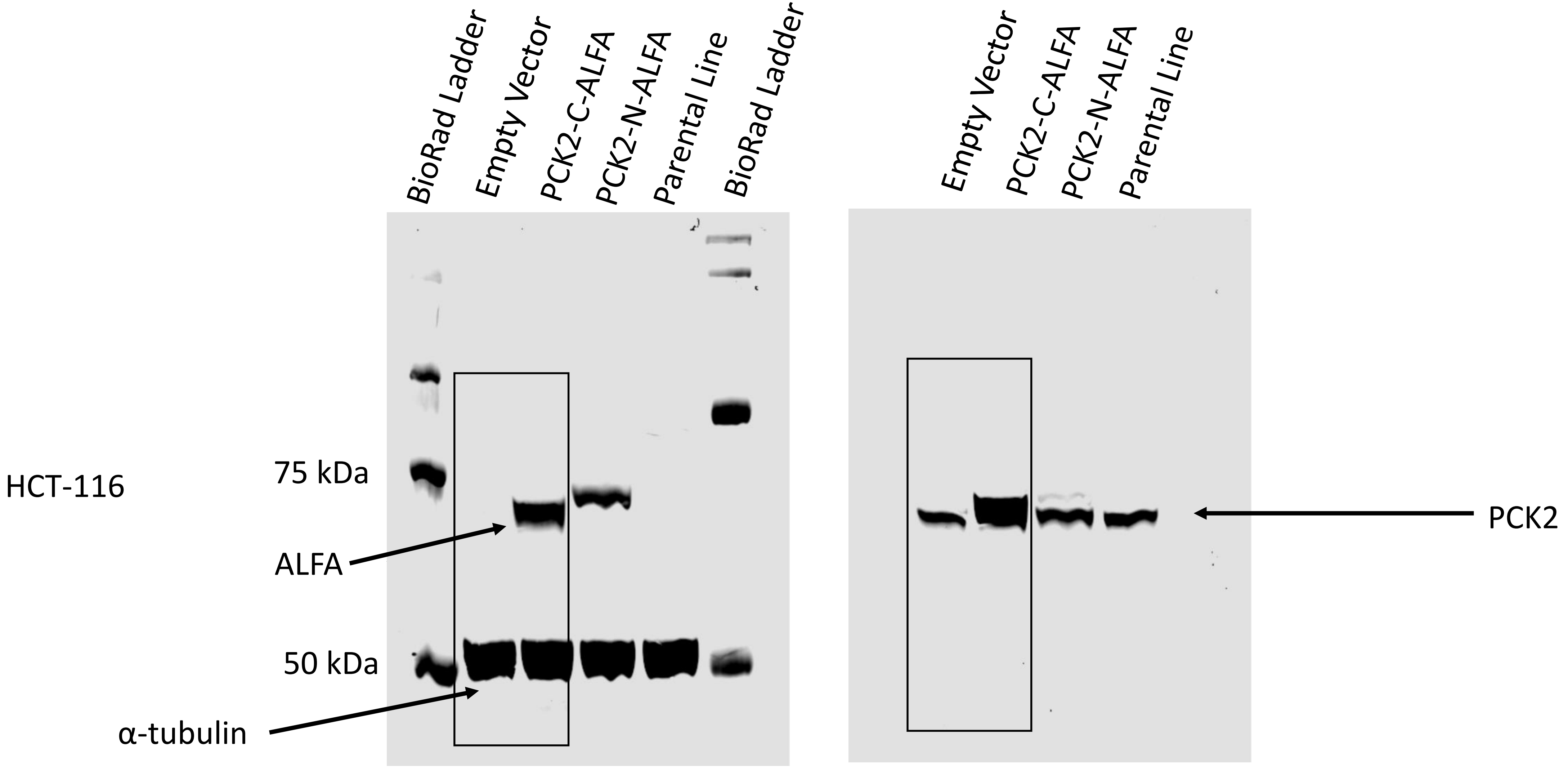

Figure S5A - Continued HT-29

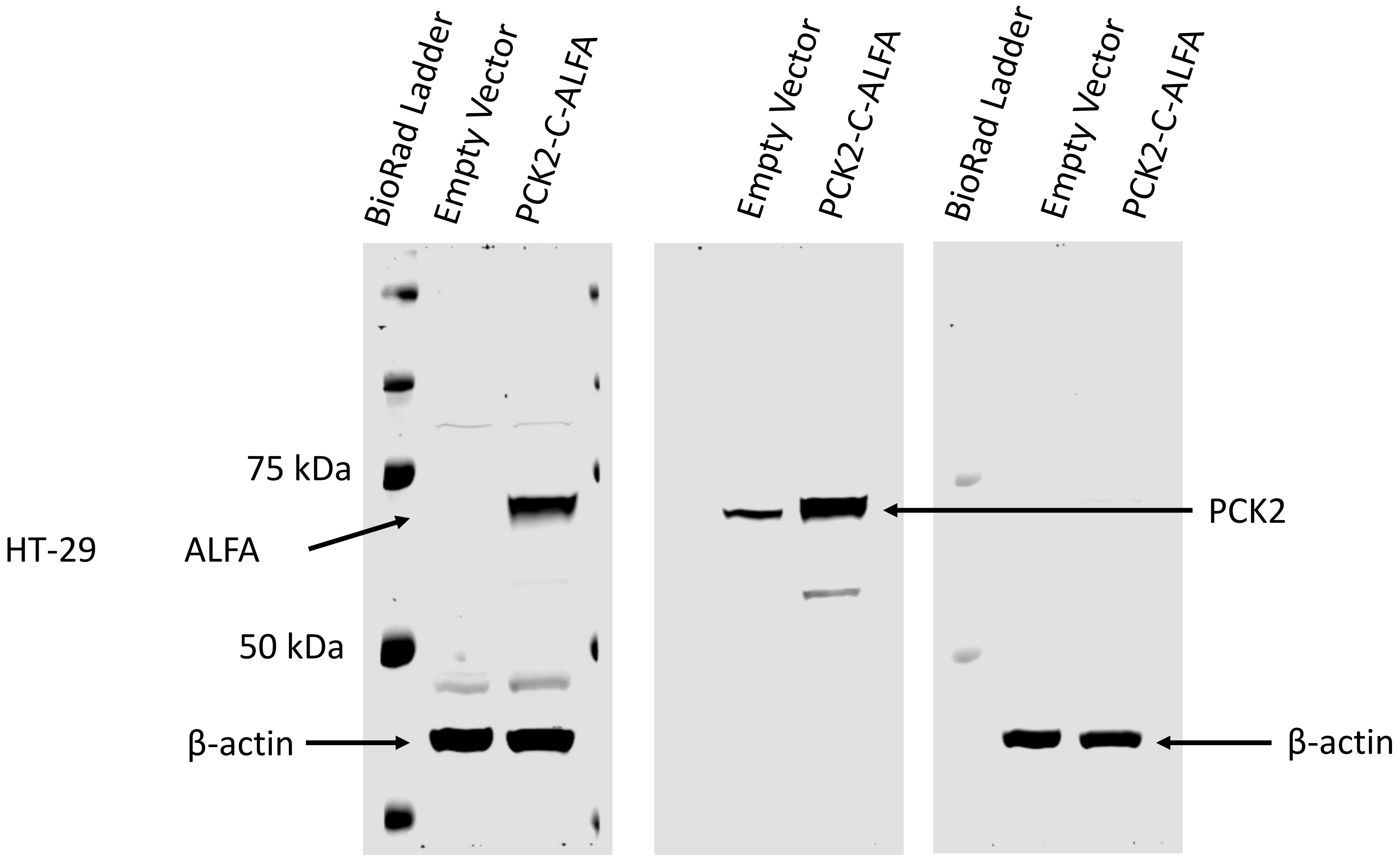

Supplemental File S6

siRNA PGC1β			siRNA ERRα		
**Gene**	**Fold Change (Log_2)**	**Adjusted P-Value**	**Gene**	**Fold Change (Log_2)**	**Adjusted P-Value**
ESRRA	-3.0501725	4.34E-23	SLC1A4	-3.8956646	2.63E-12
PCK2	-2.9644106	2.35E-13	PCK2	-3.9065517	5.96E-12
SLC1A4	-2.7414698	2.65E-12	JDP2	-2.8422453	2.00E-10
TRIB3	-1.82452	1.82E-08	LMNB1	-2.6485427	2.40E-10
PSAT1	-1.6115383	2.54E-08	CHAC1	-2.91747	6.56E-08
DDIT4	-2.0444729	4.67E-08	C1orf116	2.80287776	7.74E-08
CPA4	1.76323906	7.60E-08	HR	-2.0447284	4.57E-07
GPT2	-1.6992819	1.01E-07	MDK	-1.7412193	1.17E-06
ULBP1	-1.9837393	2.01E-07	NRP1	2.87549936	4.34E-06
ASNS	-2.4863819	4.64E-07	TFPI	1.87366954	4.55E-06
JDP2	-1.9716087	7.61E-07	PHGDH	-2.3778323	5.27E-06
IL6R	1.48849468	1.09E-06	FHL1	-2.6007623	9.10E-06
SEMA7A	1.73161535	1.17E-06	ASNS	-2.2820422	2.43E-05
KRT80	2.30875312	1.00E-05	CPA4	2.25251952	4.07E-05
NR6A1	1.70161587	1.18E-05	ZBTB20	2.25834811	8.26E-05
BIRC3	2.1938021	2.89E-05	CEACAM1	3.14424259	8.58E-05
SLC43A1	-2.8855272	3.52E-05	LMO7	2.19563308	8.58E-05
AMMECR1	1.19063512	3.58E-05	PALM3	-3.4196186	0.00010345
PDGFB	1.7233171	3.98E-05	AMPD3	1.65302827	0.00010467
LRATD2	-1.2124766	4.54E-05	SNAI2	3.16959983	0.0001191
PHGDH	-1.4993388	0.00013067	PSIP1	-1.7414462	0.0001191
STC2	-1.150869	0.00015699	BTG2	1.95825153	0.0001312
KCNH3	-1.6289723	0.00015699	KDM5B	1.50327189	0.00013924
NACC2	1.08933939	0.00015699	NUP210	-1.841356	0.00013924
SH3RF2	1.81821689	0.00036751	DOCK4	1.72009796	0.00014812
SOCS1	2.24381519	0.00036751	EIF5A2	-1.8039583	0.00014812
ANKRD52	1.32571374	0.00037372	COL17A1	2.60487877	0.0001486
PDCD4	-1.5262688	0.00053679	GPT2	-1.7892692	0.00014877
KRT23	-1.5967489	0.00061723	ZNF367	-2.5428911	0.00015907
ESRP1	-0.9707887	0.00068048	THEM6	-1.7297649	0.00017903

Genes with significant differential expression following depletion of PGC1β or ERRα compared to a non-targeting control.
